# Elucidating the Multi-Enzymatic Mechanism of Bacterial Decolorization of Azo and Indigoid Dyes: An Integrated Study of Degradation Pathways and Molecular Docking

**DOI:** 10.3390/ijms27072980

**Published:** 2026-03-25

**Authors:** Chunlei Wang, Tongshuai Liu, He Song, Yang Zhao, Haowei Wang, Jinshuo Li, Jieru Zhang, Sijia Wang, Yongdi Wang, Jixia Wang, Shumin Jiang, Chengwei Liu

**Affiliations:** 1 Key Laboratory of National Forestry and Grassland Administration on Chinese Herbal Medicine, College of Life Science, Northeast Forestry University, Harbin 150040, China; liuts012233@163.com (T.L.); 15841480554@163.com (H.S.); zy456zy789@163.com (Y.Z.); 17699399670@163.com (H.W.); ljs113619@163.com (J.L.); 15546522236@163.com (J.Z.); 13273572383@163.com (S.W.); 15588579758@163.com (Y.W.); 18846088263@163.com (J.W.); jsm9812@126.com (S.J.); 2 State Key Laboratory of Utilization of Woody Oil Resource, Northeast Forestry University, Harbin 150040, China

**Keywords:** azo dyes, biodegradation, decolorization, detoxification, molecular docking, redox enzymes

## Abstract

Synthetic dyes discharged from the textile and dyeing industry present a significant environmental and health hazard due to their inherent toxicity, environmental persistence, and potential carcinogenicity. Microbial degradation has garnered significant interest as a cost-effective and eco-friendly strategy for dye wastewater treatment in recent years. The study systematically evaluated the decolorization performance, degradation pathways, and detoxification effects of three bacterial strains, including *Rhodopseudomonas palustris* gh32, *Bacillus cereus* HL7, and *Bacillus safensis* X64, on the dye indigo carmine (IC) and three azo dyes: reactive black 5 (RB5), direct black G (DBG), and direct blue 15 (DB15). The degradation mechanisms were elucidated through UV-Vis spectroscopy, UPLC-Orbitrap-HRMS analysis, and enzyme activity assays. Molecular docking simulations were employed to investigate the interactions between key redox enzymes (such as laccase, tyrosinase, and azoreductase) and the dye molecules. The results demonstrated that the strain-specific enzymatic systems effectively disrupted the dye structures. Significant detoxification effects were further confirmed through a series of bio toxicity assays involving *Escherichia coli*, *Bacillus subtilis*, plant seeds, and erythrocytes. The addition of Fe^3+^, sodium citrate, or yeast extract significantly enhanced both the decolorization efficiency and enzyme activity. This study provides an in-depth understanding of the bacterial dye degradation process at the mechanistic level, highlighting the potential of customized bacterial systems for eco-friendly dye wastewater treatment. It offers theoretical support for elucidating the mechanisms of bacterial dye degradation and advancing bioremediation technologies.

## 1. Introduction

Synthetic dyes are essential chemical compounds extensively used in modern textile, dyeing, leather, papermaking, food, and cosmetic industries. Global annual production exceeds 700,000 t, of which approximately 10–15% is lost during production and application, eventually entering aquatic environments [1,2]. These dyes not only impart intense color to wastewater, reducing light penetration and disrupting photosynthesis in aquatic ecosystems, but also exhibit high chemical stability, biotoxicity, carcinogenicity, and mutagenicity due to their complex aromatic structures [3,4]. Among the various categories of dyes, azo dyes account for 60–70% of the total industrial dye production due to their synthetic simplicity and wide color spectrum [2,5]; indigo dyes serve as the core raw material for denim coloring and are widely utilized [6]. The azo bonds (-N=N-) or conjugated carbonyl systems within their molecular structures constitute the primary chromophores, which are also responsible for their environmental persistence and toxicity [7,8]. The discharge of ineffectively treated dye wastewater poses severe threats to aquatic organisms and human health and may lead to long-term contamination of groundwater and soil [3]. Consequently, developing efficient, economical, and environmentally friendly technologies for the removal of dye pollutants has become a pressing challenge in the field of environmental science and engineering.

Conventional physicochemical treatment methods, such as adsorption, chemical oxidation, and membrane separation, can effectively remove color from wastewater. However, they are generally limited by high operational costs and a tendency to generate secondary pollution (e.g., chemical sludge and toxic byproducts) [9,10]. In contrast, biodegradation approaches based on microorganisms and their enzymes are regarded as a highly promising green alternative technology, owing to their cost-effectiveness, environmental benignity, and mild reaction conditions [11,12]. Particularly, owing to their broad adaptability, ubiquitous distribution, and superior degradative activity, bacteria have been recognized as a key and versatile microbial group for dye degradation [13,14]. The core of microbial dye degradation lies in the secreted redox enzyme systems, including azoreductase (AZR), laccase (Lac), lignin peroxidase (Lip), tyrosinase (Tyr), and veratryl alcohol oxidase (VAO), among others. These enzymes work synergistically to specifically cleave the chromophoric groups of dyes and further break down their aromatic ring structures [15,16].

Despite extensive studies reporting the decolorization capabilities of various microorganisms toward individual dyes, critical issues in current research warrant further investigation: firstly, most existing studies have focused primarily on evaluating decolorization efficiency, while systematic, multi-level biological toxicity verification of the evolving toxicity of degradation intermediates remains insufficient [17]. Secondly, the detailed metabolic pathways by which specific bacterial strains degrade dyes of different structures, particularly the accurate identification of intermediates and pathway elucidation based on high-resolution mass spectrometry (e.g., UPLC-Orbitrap-HRMS), remain to be thoroughly elucidated [18]. Thirdly, the key enzymes driving the degradation process, along with their spatiotemporal regulatory patterns within bacterial cells, as well as the micro-scale interaction mechanisms between these enzymes and structurally diverse dye molecules, remain poorly understood. Consequently, integrated studies spanning from gene expression to protein structure are still lacking [19]. Furthermore, studies employing molecular docking techniques to elucidate, from a computational biology perspective, the differences in substrate specificity and binding affinity that account for divergent degradation efficiencies remain scarce in the field of bacterial dye degradation.

In response to the aforementioned research limitations, the novelty of this study is primarily reflected in the following three aspects: first, at the level of toxicity assessment, this study transcends the traditional single-indicator evaluation model by constructing a multi-tiered biological toxicity verification system encompassing microorganisms, plants, and mammalian cells, thereby systematically elucidating the ecotoxicological evolution patterns of dye degradation products. Second, at the mechanistic level, this study integrates high-resolution mass spectrometry analysis, subcellular-level dynamic enzyme activity monitoring, and gene expression regulation analysis to establish a comprehensive analytical framework spanning from macroscopic phenotypes to microscopic mechanisms. Third, at the molecular recognition level, this study employs homology modeling and molecular docking techniques to elucidate, from a structural biology perspective, the substrate-specific recognition mechanisms of oxidoreductases toward structurally diverse dye molecules, providing a novel molecular basis for explaining strain-specific substrate preferences and differential degradation efficiencies. Such multi-dimensional, cross-scale systematic investigations remain relatively rare in the field of bacterial dye degradation.

To systematically address the aforementioned issues, this study focuses on three bacterial strains with potential dye-degrading capabilities: *Rhodopseudomonas palustris* gh32, *Bacillus cereus* HL7, and *Bacillus safensis* X64, and investigates the following core scientific questions: (1) Do different bacterial strains exhibit significant differences in their decolorization capabilities toward indigoid and azo dyes, and how do exogenous additives regulate their decolorization efficiency? (2) Through which metabolic pathways do these strains progressively degrade complex dye molecules, and how do the intermediate products evolve during the degradation process? (3) Does biodegradation genuinely achieve “detoxification,” and how do the toxic effects of dyes and their degradation products on microorganisms, plants, and mammalian cells change? (4) Which key oxidoreductases are involved in the degradation process, what are their spatiotemporal distribution and activity dynamics within and outside cells, and how does their gene expression respond to dye stress? (5) How is specific recognition between enzymes and dye molecules achieved, and what is the molecular basis for the differential binding affinities of oxidoreductases from different sources toward structurally diverse dyes? By addressing these questions, this study aims to establish a comprehensive cognitive framework spanning from macroscopic decolorization performance to microscopic molecular mechanisms, thereby providing systematic experimental evidence and theoretical interpretation for an in-depth understanding of the complex mechanisms underlying bacterial dye degradation.

## 2. Results

### 2.1. Bacterial Decolorization Capability on Azo Dyes and Indigoid Dyes

To investigate the decolorization capability of the strains *Rhodopseudomonas palustris* gh32, *Bacillus cereus* HL7, and *Bacillus safensis* X64 toward IC, RB5, DBG, and DB15, the absorbance values at the maximum absorption wavelength of each dye were measured before and after biological treatment. The decolorization efficiency was used to evaluate the dye removal performance of each strain. The results are presented in Table 1. Strain gh32 exhibited decolorization activity toward IC and RB5, strain HL7 toward DBG, strain X64 toward DB15, and strain HL7 also showed activity toward IC. The addition of FeCl_3_ significantly enhanced the decolorization efficiency of strain HL7 for IC. Similarly, supplementing with sodium citrate and yeast extract as substrates also led to a significant increase in the decolorization rate of DB15 by strain X64. This observation aligns with previous studies, where the addition of exogenous cofactors such as Fe^3+^ can markedly enhance the activity of bacterial oxidoreductases, thereby improving decolorization performance [20]. Similarly, the supplementation of sodium citrate or yeast extract as readily utilizable carbon sources or redox mediators has been shown to promote microbial growth and enhance enzymatic activity, thereby improving dye decolorization performance [21,22,23].

### 2.2. Mechanisms of Dye Decolorization by Bacterial Strains

#### 2.2.1. Scanning Spectrophotometric Analysis

After microbial treatment, although visible decolorization of the dyes and high decolorization rates were obtained, it remains necessary to analyze whether decolorization occurred via adsorption or degradation by examining the full-wavelength UV-Vis scanning spectra of the dyes and their potential degradation products. If the characteristic absorption peaks of the dye show only a reduction in intensity while their positions and shapes remain largely unchanged, this may indicate adsorption of dye molecules onto the cell surface, leading to a decrease in dye concentration in the solution. In contrast, if significant changes are observed in the characteristic absorption peaks, such as peak shifts, alterations in peak shape, attenuation, or disappearance of original peaks. This suggests that the chromophoric structure of the dye molecules has been disrupted, indicating degradation. Furthermore, the emergence of new absorption peaks signifies the formation of new degradation products.

**Figure 1:**
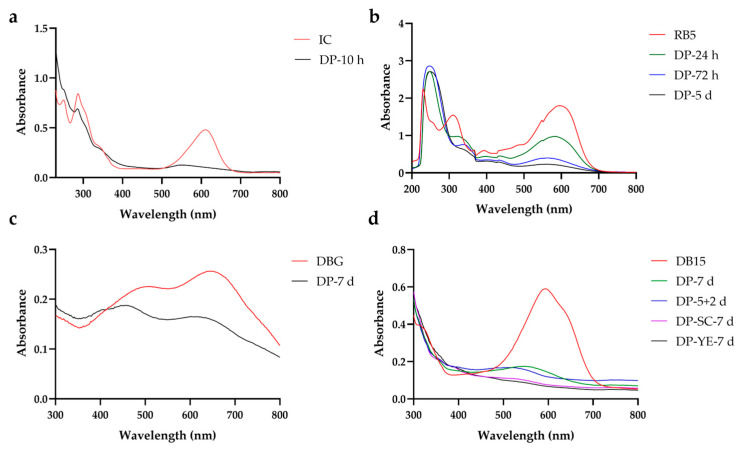
UV-Vis absorption spectra of dyes before and after treatment by strains. (**a**) IC by strain gh32; (**b**) RB5 by strain gh32; (**c**) DBG by strain HL7; and (**d**) DB15 by strain X64. DP: degradation product; DP-10 h: DP after 10 h of agitation; DP-24 h/72 h/5 d: DP after 24 h/72 h/5 d of static treatment; DP-7 d: DP after 7 d of static treatment with sealing film; DP-5 + 2 d: DP after 5 d of static treatment with sealing film followed by 2 d of agitation; DP-SC-7 d: DP after 7 d of static treatment with sealing film added sodium citrate; DP-YE-7 d: DP after 7 d of static treatment with sealing film added yeast extract.

IC exhibits characteristic absorption peaks in the ultraviolet region at 288 nm and in the visible region at 610 nm. After treatment with strain gh32 for 10 h, the peak at 610 nm disappeared, and a new absorption peak emerged at 315 nm (Figure 1a). This indicates that the core chromophoric structure of IC was cleaved, specifically the conjugated carbon-carbon double bond (-C=C-) that links the two carbonyl groups. As a result, its extended conjugated system was disrupted, leading to the loss of characteristic absorption in the visible region and generating two or more colorless or lightly colored intermediate metabolites containing aromatic ring structures.

RB5 exhibits characteristic absorption peaks in the ultraviolet region at 310 nm and in the visible region at 596 nm. After treatment with strain gh32, the characteristic peak at 596 nm gradually weakened and eventually disappeared with prolonged treatment time, while a new absorption peak emerged at 248 nm (Figure 1b). This indicates that the azo bond of RB5, along with the conjugated chromophoric structure formed by its linked naphthalene and benzene rings, was disrupted. As a result, the dye was decolorized, yielding small-molecule degradation intermediates containing aromatic or naphthalene ring structures.

DBG exhibits characteristic absorption peaks at 508 nm and 648 nm. After treatment with strain HL7, a significant decrease in the intensity of these characteristic peaks was observed, accompanied by a distinct blue shift (Figure 1c). This indicates that the azo bond and the conjugated system of the linked benzene/naphthalene rings within the DBG molecule were disrupted. The resultant reduction in the conjugated structure led to a shorter absorption wavelength, thereby achieving decolorization and generating corresponding intermediate metabolites.

DB15 displays a characteristic absorption peak at 594 nm. Following treatment with strain X64, the intensity of this characteristic peak significantly decreased, nearly disappearing, and was accompanied by a blue shift phenomenon. Its changing trend is similar to the degradation process of DBG. Compared to the treatment group without supplemented substrates, the addition of sodium citrate (SC) or yeast extract (YE) significantly enhanced the degradation capability of strain X64 toward DB15. Furthermore, a comparison of treatment methods revealed that a sequential approach involving 5 d of sealed static incubation followed by 2 d of shaking was markedly more effective in degradation than continuous sealed static incubation for 7 d (Figure 1d).

#### 2.2.2. Identification of Degradation Intermediates Using UPLC-Orbitrap-HRMS and Development of Degradation Pathway Diagrams

**Figure 2:**
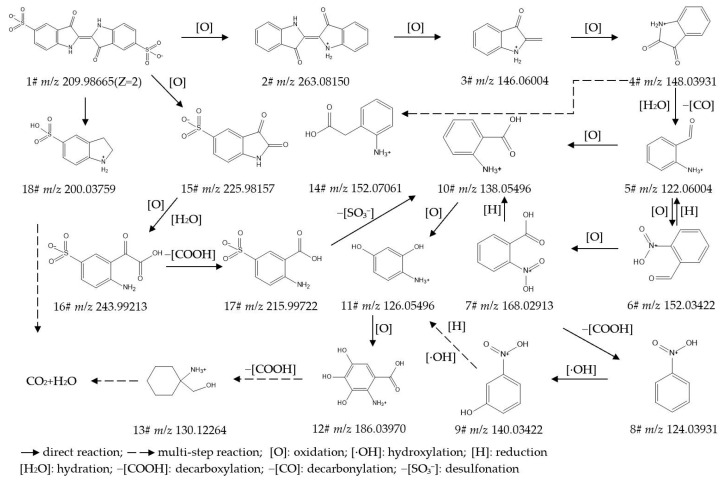
Degradation pathway of IC by strain gh32. The *m*/*z* values shown in the figure are theoretical values.

**Figure 3:**
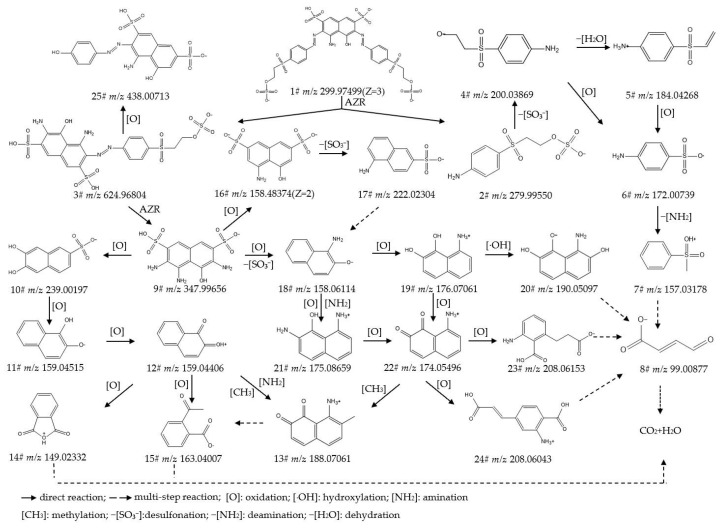
Degradation pathway of RB5 by strain gh32. The *m*/*z* values shown in the figure are theoretical values. AZR: azo reductase.

**Figure 4:**
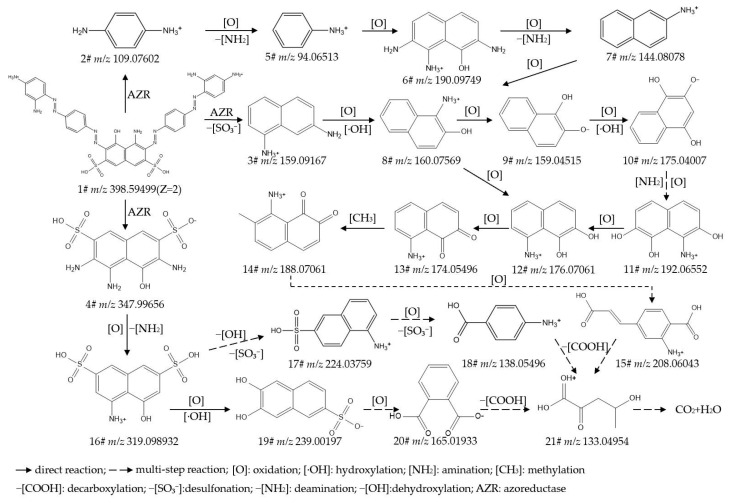
Degradation pathway of DBG by strain HL7. The *m*/*z* values shown in the figure are theoretical values. AZR: azo reductase.

**Figure 5:**
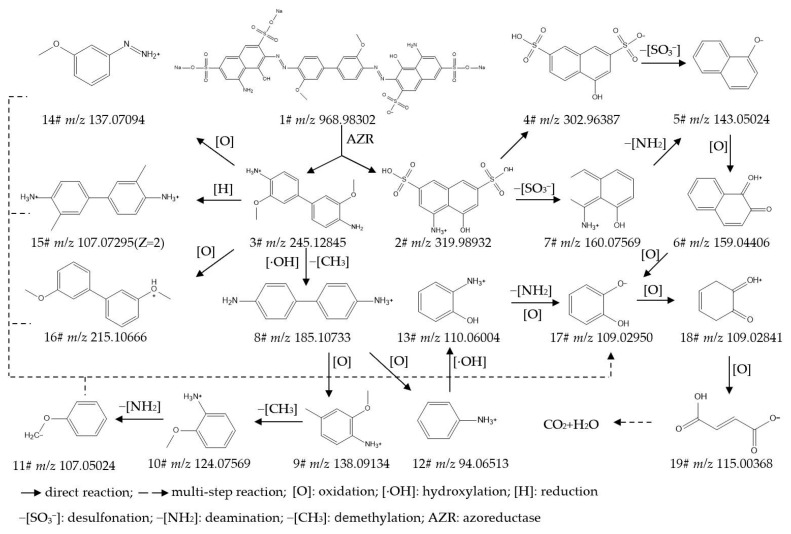
Degradation pathway of DB15 by strain X64. The *m*/*z* values shown in the figure are theoretical values. AZR: azo reductase.

To identify the intermediate metabolites generated from the biodegradation of IC, RB5, DBG, and DB15, the data acquired by UPLC-Orbitrap-HRMS (liquid chromatography: Vanquish; mass spectrometer: Q Exactive Focus) under both positive- and negative-ion electrospray ionization (ESI) modes were analyzed, leading to the detection of multiple intermediate metabolites. The intermediate metabolites generated from the degradation of the respective dyes were characterized based on retention time, theoretical/experimental mass-to-charge ratio (*m*/*z*), and adduct forms. Tables S1, S2, S4 and S6 provide detailed listings of the chemical formula, compound name, molecular weight (mol wt.), adduct of the substance, experimental *m*/*z* of the adduct, theoretical *m*/*z* of the adduct, difference in *m*/*z*, retention time, and CAS/CID for the original dyes and their degradation intermediates, where Compound **1#** in each supplementary table corresponds to the parent dye. Figures S1–S4 present detailed mass spectra of the original dyes and their biodegradation intermediates for IC, RB5, DBG, and DB15, respectively. The proposed biodegradation pathways for these four dyes are illustrated in Figure 2, Figure 3, Figure 4 and Figure 5.

##### Analysis and Proposed Degradation Pathway of IC

By tracking and analyzing the products generated during IC degradation using UPLC-Orbitrap-HRMS, 17 major intermediates were successfully identified (details provided in Table S1), and a plausible degradation pathway for IC was proposed accordingly (Figure 2).

1. Oxidative Initiation: Lignin peroxidase (Lip) and veratryl alcohol oxidase (VAO) attack IC (**1#**, *m*/*z* 209.98665), directly removing the sulfonic acid group to generate (2E)-2-(3-oxo-1H-indol-2-ylidene)-1H-indol-3-one (**2#**, *m*/*z* 263.08150). This product is further oxidized to 2-methylidene-1H-indol-3-one (**3#**, *m*/*z* 146.06004) and isatin (**4#**, *m*/*z* 148.03931). Concurrently, Lip attacks the core structure of IC (**1#**, *m*/*z* 209.98665), cleaving the C=C bond via oxidation to yield sulfonated aromatic intermediates such as 2,3-dioxo-5-indolinesulfonic acid (**15#**, *m*/*z* 225.98157). The formation of Compound **15#** signifies the cleavage of the trans-2,2′-biindolinylidene-3,3′-dione chromophoric core of IC.
2. Ring Cleavage and Decarboxylation/Desulfonation Reactions: The indole ring undergoes ring cleavage via hydrolysis, decarboxylation, decarbonylation, and desulfonation, leading to the formation of a series of benzene derivatives. These include compounds such as 2-(2-amino-5-sulfophenyl)-2-oxoacetic acid (**16#**, *m*/*z* 243.99213), 2-amino-5-sulfobenzoic acid (**17#**, *m*/*z* 215.99722), 2-aminobenzoic acid (**10#**, *m*/*z* 138.05496), 2-aminophenylacetic acid (**14#**, *m*/*z* 152.07061), and 2-aminobenzaldehyde (**5#**, *m*/*z* 122.06004). Notably, the desulfonation reaction converts the recalcitrant sulfonated aromatic acid (**17#**) into more readily degradable aromatic acids (**10#**), thereby facilitating subsequent metabolic steps.
3. Functional Group Reduction and Detoxification: Under the action of reductases, nitro-group-containing (-NO_2_) toxic intermediates—such as 2-nitrobenzaldehyde (**6#**, *m*/*z* 152.03422), 2-nitrobenzoic acid (**7#**, *m*/*z* 168.02913), nitrobenzene (**8#**, *m*/*z* 124.03931), and 3-nitrophenol (**9#**, *m*/*z* 140.03422)—are reduced to their corresponding amino-group-containing (-NH_2_) compounds, exemplified by 4-aminobenzene-1,3-diol (**11#**, *m*/*z* 126.05496). This conversion significantly reduces the toxicity of the nitro-substituted intermediates.
4. Dearomatization: Catalyzed by tyrosinase, Compound **11#** is converted into 2-amino-3,4,5-trihydroxybenzoic acid (**12#**, *m*/*z* 186.03970). This transformation deeply activates its aromatic ring. Subsequent reactions, including decarboxylation, lead to the formation of the alicyclic compound (1-aminocyclohexyl)methanol (**13#**, *m*/*z* 130.12264). This alicyclic intermediate can undergo hydrolytic ring-opening, subsequently entering pathways such as fatty acid β-oxidation and the tricarboxylic acid (TCA) cycle.

##### Analysis and Proposed Degradation Pathway of RB5

Samples from the treatment of RB5 by strain gh32 for 24 h and 5 d were analyzed by UPLC-Orbitrap-HRMS. A total of 24 major intermediate products were identified (details are provided in Table S2). Differences in the degradation products generated between the two time points were compared (Table S3), and a plausible degradation pathway for RB5 was proposed based on these findings (Figure 3).

1. Azo Bond Reductive Cleavage and Initial Fragmentation: The degradation of RB5 (**1#**, *m*/*z* 299.97499) is initiated by the catalytic action of azoreductase (AZR). The azo bond (-N=N-) within its molecular structure undergoes specific reductive cleavage. This generates initial fragments, typified by compounds such as 2-[(4-aminophenyl)sulfonyl]ethyl hydrogen sulfate (**2#**, *m*/*z* 279.99500) and 3,5-diamino-4-hydroxy-6-[[4-(2-sulfooxyethylsulfonyl)phenyl]diazenyl]naphthalene-2,7-disulfonic acid (**3#**, *m*/*z* 624.96804). This step signifies the depolymerization and decolorization of the dye molecule’s extensive, linear, and highly conjugated π-electron delocalization system, which is constructed from the “azo bond + naphthalene ring + benzene ring” framework.
2. Naphthalene Ring Oxidation, Hydroxylation, and Functional Group Simplification: The naphthalene-based intermediate 3,4,6-triamino-5-hydroxynaphthalene-2,7-disulfonic acid (**9#**, *m*/*z* 347.99656) undergoes desulfonation and deamination catalyzed by tyrosinase (Tyr) to yield 6,7-dihydroxynaphthalene-2-sulfonic acid (**10#**, *m*/*z* 239.00197). Alternatively, within the bacterial cells, oxidative enzymes can catalyze its deamination to produce disodium;4-amino-5-hydroxynaphthalene-2,7-disulfonate (**16#**, *m*/*z* 158.48374). Furthermore, 1-amino-2-naphthol (**18#**, *m*/*z* 158.06114) is converted by Tyr into hydroxylated naphthalene derivatives, such as 8-aminonaphthalene-1,2-diol (**19#**, *m*/*z* 176.07061). This compound is subsequently oxidized to quinone-type naphthalene derivatives, exemplified by 8-aminonaphthalene-1,2-dione (**22#**, *m*/*z* 174.05496).
3. Aromatic Ring Cleavage and Formation of Small-Molecule Carboxylic Acids: Quinonoid naphthalene structures, such as naphthalene-1,2-dione (**12#**, *m*/*z* 159.04406) and 8-aminonaphthalene-1,2-dione (**22#**, *m*/*z* 174.05496), undergo ring-cleavage reactions catalyzed by strong oxidative enzymes, including lignin peroxidase (Lip) and veratryl alcohol oxidase (VAO). This process generates various ring-opened products, such as 2-amino-6-(2-carboxyethyl)benzoic acid (**23#**, *m*/*z* 208.06153), 2-acetylbenzoic acid (**15#**, *m*/*z* 163.04007), and 2-benzofuran-1,3-dione (**14#**, *m*/*z* 149.02332). Consequently, the aromatic ring structures are further disrupted and converted into aliphatic carboxylic acid chains. Small-molecule carboxylic acids, such as 4-oxobut-2-enoic acid (**8#**, *m*/*z* 99.00877), along with Compounds **14#** and **15#**, can subsequently enter central metabolic pathways, including β-oxidation and the tricarboxylic acid (TCA) cycle.

##### Analysis and Proposed Degradation Pathway of DBG

Samples from the treatment of DBG by strain HL7 for 7 d, by strain HL7 with the mediator anthraquinone-2-sulfonate for 5 d, and by purified azoreductase for 2 d were analyzed using UPLC-Orbitrap-HRMS. A total of 20 major intermediate products were identified (details are provided in Table S4), and differences in the degradation products generated among the various treatments were compared (Table S5).

Based on these findings, a plausible degradation pathway for DBG was proposed (Figure 4).

1. Initial Attack and Chromophore Destruction: DBG (**1#**, *m*/*z* 398.59499) is initially attacked by azoreductase (AZR), which catalyzes the reductive cleavage of the azo bond (-N=N-) within its molecular structure. This cleavage generates fragments, including p-phenylenediamine (**2#**, *m*/*z* 109.07602), 1,7-naphthalenediamine (**3#**, *m*/*z* 159.09167), and 3,4,6-triamino-5-hydroxynaphthalene-2,7-disulfonic acid (**4#**, *m*/*z* 347.99656). In DBG, the conjugated aromatic rings and the azo bonds linking them collectively form an extensive π-conjugated system spanning the entire molecule. The cleavage of the azo bond disrupts this conjugated system, thereby achieving decolorization.
2. Ring-Opening Reactions and Formation of Aromatic Intermediates: The generated aromatic amine intermediates are further acted upon by oxidases such as laccase (Lac), lignin peroxidase (Lip), and tyrosinase (Tyr). These enzymes mediate reactions, including hydroxylation and oxidative ring-cleavage, converting the intermediates into a series of structurally simpler and more polar small molecule benzene- and naphthalene-based derivatives, such as phthalic acid (**20#**, *m*/*z* 165.01933), 4-aminobenzoic acid (**18#**, *m*/*z* 138.05496), and naphthalene-1,2-diol (**9#**, *m*/*z* 159.04515).
3. Structural Evolution and Toxicity Reduction: As the degradation proceeds, the complex polycyclic structure of DBG is progressively broken down, yielding a series of small-molecule aromatic and aliphatic acids. For instance, the more toxic intermediate 1-amino-2-naphthol (**8#**, *m*/*z* 160.07569) is oxidized by veratryl alcohol oxidase (VAO) and tyrosinase (Tyr) to 8-aminonaphthalene-1,2-dione (**13#**, *m*/*z* 174.05496). This quinone subsequently undergoes ring cleavage, forming the less toxic compound 2-amino-4-(2-carboxyethenyl)benzoic acid (**15#**, *m*/*z* 208.06043). The benzene-series intermediates undergo further steps such as ring cleavage and decarboxylation, generating aliphatic organic acids, including 4-hydroxy-2-oxopentanoic acid (**21#**, *m*/*z* 133.04954).

##### Analysis and Proposed Degradation Pathway of DB15

Samples of strain X64 treated with DB15 under four conditions, including static incubation for 7 d, static incubation for 5 d followed by agitation for 2 d, supplementation with yeast extract for 7 d, and supplementation with sodium citrate for 7 d, were analyzed by UPLC-Orbitrap-HRMS. This led to the successful identification of 18 major intermediate metabolites (details provided in Table S6). Differences in the degradation products generated among the various treatments were compared (Table S7), and a plausible degradation pathway for DB15 was proposed based on these findings (Figure 5).

1. Initial Attack and Chromophore Destruction: DB15 (**1#**, *m*/*z* 968.98302) is initially attacked by azoreductase (AZR), which catalyzes the reductive cleavage of the azo bonds (-N=N-). This results in the generation of 4-amino-5-hydroxy-2,7-naphthalenedisulfonic acid (**2#**, *m*/*z* 319.98932) and 3,3′-dimethoxybenzidine (**3#**, *m*/*z* 245.12845). Decolorization is achieved in this step through the direct cleavage of the bis-azo-benzidine bridge, which disrupts the dye’s essential chromophoric conjugated system.
2. Ring-Opening Reactions and Formation of Benzene Series Compounds: The aforementioned intermediates are further acted upon by enzymes, including laccase (Lac), lignin peroxidase (Lip), tyrosinase (Tyr), and desulfonases. These catalyze reactions such as naphthalene ring cleavage, desulfonation, and oxidation, leading to the formation of a series of small-molecule benzene-series derivatives. Examples include 1-naphthol (**5#**, *m*/*z* 143.05024), aniline (**12#**, *m*/*z* 94.06513), and catechol (**17#**, *m*/*z* 109.02950).
3. Structural Evolution and Toxicity Transformation of Degradation Products: As the degradation proceeds, the complex azo structure of DB15 is progressively converted into low-toxicity or non-toxic small molecules. For instance, under the action of oxidases and hydroxylases, more toxic benzidine-type intermediates, such as benzidine (**8#**, *m*/*z* 185.10733), are transformed into hydroxylated products such as 2-aminophenol (**13#**, *m*/*z* 110.06004). This structural change demonstrates the synergistic detoxification capability of the multi-enzyme system. The detection of a fatty aliphatic organic acid, but-2-enedioic acid (**19#**, *m*/*z* 115.00368), indicates that the benzene-series intermediates have undergone thorough fragmentation via steps such as ring-cleavage and desulfonation.

#### 2.2.3. Detoxification Evaluation of Biodegraded Dye Products

Microbial azoreductase cleaves azo bonds and generates aromatic amines such as β-naphthylamine, aniline, and p-phenylenediamine, several of which have been classified by the International Agency for Research on Cancer (IARC) as confirmed human carcinogens [24,25]. The degradation of contaminants does not equate to the elimination of their toxicity; on the contrary, this process can lead to the generation of byproducts with persistent hazardous effects [26]. To verify whether genuine “detoxification” was achieved following the biodegradation of the dyes, this study evaluated the ecotoxicity of the dyes and their degradation products at three levels: microbial, plant seed, and mammalian cell.

##### Assessment of Biodegradation and Detoxification of IC by Strain gh32

**Figure 6:**
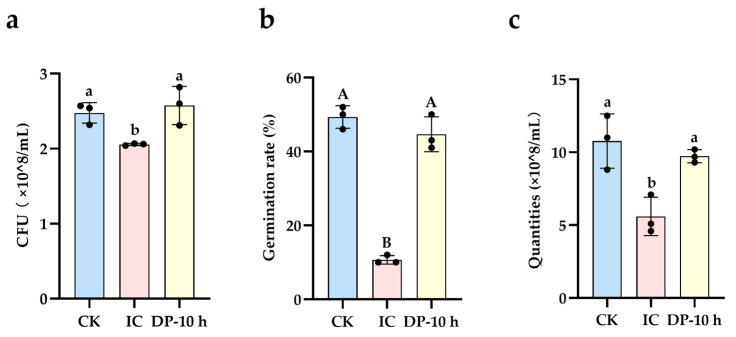
Biotoxicity of dye IC and its degradation products on (**a**) *E. coli*, (**b**) tobacco seeds, and (**c**) normal erythrocytes by *R. palustris* gh32. DP: degradation product; DP-10 h: DP after 10 h of agitation. In figure (**a**), PBS buffer (pH 7.0) was used as the CK control. In figure (**b**), sterile water was used as the CK control. In figure (**c**), normal saline was used as the CK control. The data are analyzed by one-way ANOVA with a Tukey multiple comparisons test using means of three experiments. Different lowercase letters indicate significant difference at *p* < 0.05. Different uppercase letters indicate significant difference at *p* < 0.01.

Compared to the parent IC, the degradation products (DP-10 h) generated by strain gh32 showed a significant reduction in their inhibitory effect on *E. coli* growth, with no significant difference from the control group (Figure 6a). While the parent IC severely inhibited tobacco seed germination, the germination rate in the degradation product group was not significantly different from that of the control (Figure 6b). The parent IC caused significant hemolysis of red blood cells, whereas the degradation product group showed no significant difference from the control (Figure 6c). These results confirm that the toxicity of dye IC toward *E. coli*, tobacco seeds, and red blood cell membranes was effectively eliminated following treatment with strain gh32. The degradation process mediated by strain gh32 not only achieved decolorization but also efficiently removed the toxicity of IC across different biological levels, resulting in genuine biodetoxification. The decrease in toxicity corresponds to the structural characteristics of the products, namely the disappearance of the indigo core and an increased proportion of amino-/hydroxy-substituted compounds (Figure 2), further validating the effectiveness of the enzymatic degradation.

##### Assessment of Biodegradation and Detoxification of RB5

**Figure 7:**
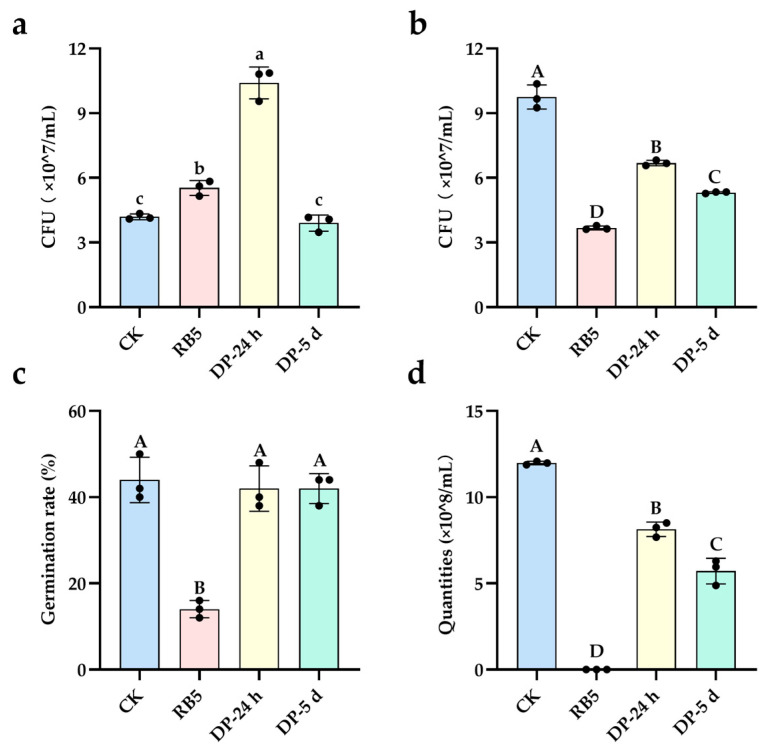
Biotoxicity of dye RB5 and its degradation products on (**a**) *E. coli*, (**b**) *B. subtilis*, (**c**) tobacco seeds, and (**d**) normal erythrocytes by *R. palustris* gh32. DP: degradation product; DP-24 h/5 d: DP after 24 h/5 d of static treatment. In figure (**a**,**b**), PBS buffer (pH 7.0) was used as the CK control. In figure (**c**), sterile water was used as the CK control. In figure (**d**), normal saline was used as the CK control. The data are analyzed by one-way ANOVA with a Tukey multiple comparisons test using means of three experiments. Different lowercase letters indicate significant difference at *p* < 0.05. Different uppercase letters indicate significant difference at *p* < 0.01.

Compared to the control group (CK), both the parent RB5 dye and its 24-h degradation products (DP-24 h) significantly promoted the growth of *E. coli*; however, the degradation products after 5 d showed no significant difference from the CK group (Figure 7a), indicating that *E. coli* could utilize RB5 and its degradation intermediates for growth. Relative to the parent RB5, the degradation products (DP-24 h and DP-5 d) generated by strain gh32 exhibited an extremely significant growth-promoting effect on *Bacillus subtilis*, although the colony-forming units in both degradation product groups remained significantly lower than those in the control group (Figure 7b). This suggests that RB5 possessed biotoxicity toward *B. subtilis*, which was effectively detoxified following degradation by strain gh32. Compared to the parent RB5, the degradation products (DP-24 h and DP-5 d) from strain gh32 extremely significantly increased the germination rate of tobacco seeds, with no statistically significant difference from the control group (Figure 7c), demonstrating that RB5 was toxic to tobacco seeds but that this toxicity was eliminated after treatment with strain gh32. In contrast to the parent RB5, the degradation products (DP-24 h and DP-5 d) from strain gh32 significantly increased the number of intact (non-lysed) red blood cells, although the values remained significantly lower than those in the control group (Figure 7d). This indicates that RB5 exerted membrane toxicity on red blood cells, but its toxicity was alleviated after degradation by strain gh32.

##### Assessment of Biodegradation and Detoxification of DBG

**Figure 8:**
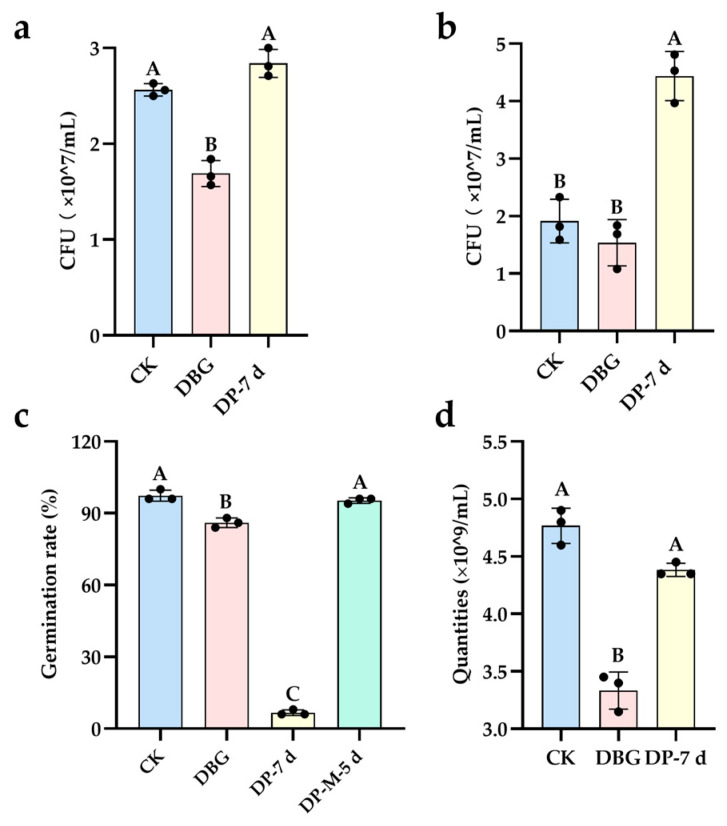
Biotoxicity of dye DBG and its degradation products on (**a**) *E. coli*, (**b**) *B. subtilis*, (**c**) *P. radiatus* seeds, and (**d**) normal erythrocytes by *B. cereus* HL7. DP: degradation product; DP-7 d: DP after 7 d of static treatment with sealing film; DP-M-5 d: DP after 5 d by static treatment with sealing film added sodium 2-anthraquinonesulfonate as redox mediator. In figure (**a**,**b**), PBS buffer (pH 7.0) was used as the CK control. In figure (**c**), sterile water was used as the CK control. In figure (**d**), normal saline was used as the CK control. The data are analyzed by one-way ANOVA with a Tukey multiple comparisons test using means of three experiments. Different uppercase letters indicate significant difference at *p* < 0.01.

Compared to the parent DBG, the degradation products (DP-7 d) generated by strain HL7 showed a statistically significant promotion of *E. coli* growth, with no significant difference from the control group (Figure 8a). This indicates that DBG exhibited biotoxicity toward *E. coli*, which was effectively eliminated after treatment with strain HL7. No significant difference was observed between the parent DBG group and the control group. In contrast, the degradation product group significantly increased the colony-forming units (CFU) of *Bacillus subtilis* (Figure 8b), suggesting that DBG was non-toxic to *B. subtilis*, and its degradation products could promote its growth. Relative to the control, the parent DBG significantly inhibited the germination of mung bean seeds. While the degradation products alone did not eliminate this toxicity, supplementation with the mediator anthraquinone-2-sulfonate followed by treatment with strain HL7 resulted in a significant increase in germination rate, showing no significant difference from the control group (Figure 8c). This demonstrates that the products from this mediator-mediated redox reaction successfully removed DBG’s toxicity to mung bean seeds. DBG was toxic to red blood cells, causing a significant decrease in their count, whereas its degradation products significantly improved intact (non-lysed) red blood cell survival, showing no significant difference from the control (Figure 8d).

##### Assessment of Biodegradation and Detoxification of DB15

**Figure 9:**
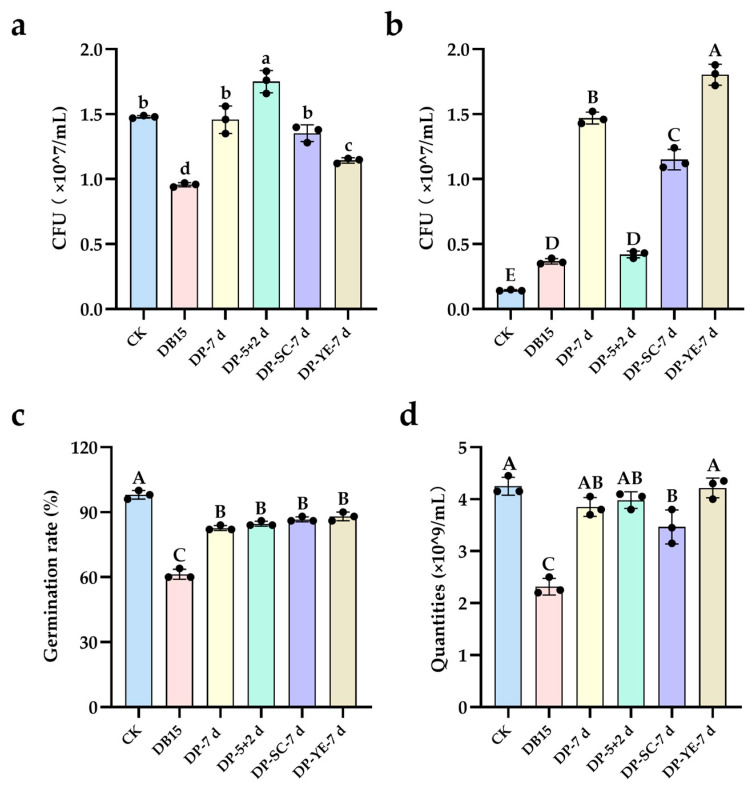
Biotoxicity of dye DB15 and its degradation products on (**a**) *E. coli*, (**b**) *B. subtilis*, (**c**) *P. radiatus* seeds, and (**d**) normal erythrocytes by *B. safensis* X64. DP: degradation product; DP-7 d: DP after 7 d of static treatment with sealing film; DP-5 + 2 d: DP after 5 d of static treatment with sealing film followed by 2 d of agitation; DP-SC-7 d: DP after 7 d of static treatment with sealing film added sodium citrate; DP-YE-7 d: DP after 7 d of static treatment with sealing film added yeast extract. In figure (**a**,**b**), PBS buffer (pH 7.0) was used as the CK control. In figure (**c**), sterile water was used as the CK control. In figure (**d**), normal saline was used as the CK control. The data are analyzed by one-way ANOVA with a Tukey multiple comparisons test using means of three experiments. Different lowercase letters indicate significant difference at *p* < 0.05. Different uppercase letters indicate significant difference at *p* < 0.01.

Compared to the parent DB15, the degradation products obtained after treatment with strain X64 significantly promoted the growth of *E. coli*. These products showed no significant difference from the control group for the DP-7 d and DP-SC-7 d treatments, while a significant growth promotion was observed for the DP-5 + 2 d treatment (Figure 9a). This indicates that the treatment with strain X64 removed the toxicity of DB15 toward *E. coli*. Furthermore, the degradation products generated from the sequential treatment of static incubation for 5 d followed by shaking for 2 d exhibited even lower toxicity and could be utilized by *E. coli* for growth. Compared to the control, both the parent DB15 and its degradation products significantly promoted the growth of *Bacillus subtilis* (Figure 9b), suggesting that *B. subtilis* can metabolize the dye and its degradation intermediates as carbon or nitrogen sources. Compared to the parent DB15, the degradation products generated by strain X64 led to an extremely significant increase in the germination rate of mung bean seeds, although the rates remained significantly lower than those in the control group (Figure 9c). This indicates that DB15 exhibited biotoxicity toward mung bean seeds, which was mitigated following degradation by strain X64. In contrast to the parent DB15, the degradation products obtained after treatment with strain X64 significantly increased the number of intact (non-lysed) red blood cells, showing no significant difference from the control group for the DP-7 d, DP-5 + 2 d, and DP-YE-7 d treatments (Figure 9d). This demonstrates that the treatment with strain X64 completely eliminated the toxicity of dye DB15 toward red blood cells.

#### 2.2.4. Dye-Induced Changes in Oxidoreductase Activity Within Biological Systems

To elucidate the enzymatic driving mechanisms underlying the aforementioned degradation process, the activity changes of key redox enzymes in the strains under dye stress, as well as the toxicity changes of the dyes and their degradation products, were determined. Combined with the results from biotoxicity studies, the mechanisms of dye degradation were comprehensively analyzed. Under IC induction, both cell surface and intracellular activities of veratryl alcohol oxidase (VAO) (Figure S5a) and lignin peroxidase (Lip) (Figure S5b) in strain R. palustris gh32 increased significantly to varying degrees. The cell surface tyrosinase (Tyr) activity exhibited a highly significant increase (Figure S5c). These elevated enzyme activities closely coincided with the phase of rapid IC concentration decline, indicating that they serve as key catalysts for the initial oxidative cleavage of IC.

IC exhibits low acute toxicity, while its degradation product nitrobenzene (**8#**, *m*/*z* 124.03931) is highly toxic, capable of causing hematotoxicity and liver damage. In the degradation pathway, nitrobenzene can be converted into the less toxic 3-nitrophenol ( **9#**, *m*/*z* 140.03422), which is subsequently oxidized by Tyr to 2-amino-3,4,5-trihydroxybenzoic acid (**12#**, *m*/*z* 186.03970) (Table S1). Biotoxicity test results also confirmed that the toxicity of dye IC was effectively eliminated by strain gh32, showing no significant difference compared to the control (Figure 6).

Under RB5 stress, the dynamic activity changes of multiple key degradation enzymes in strain gh32 are shown in Figure S6. The activities of oxidative enzyme systems, including veratryl alcohol oxidase (VAO) (Figure S6a), lignin peroxidase (Lip) (Figure S6b), and tyrosinase (Tyr) (Figure S6c), as well as reductase systems such as azoreductase (AZR) (Figure S6d) and riboflavin reductase (RR) (Figure S6e), were significantly induced in different cellular compartments.

The subcellular localization analysis of enzymes revealed that during the 0–24 h decolorization phase, intracellular Tyr activity, extracellular RR and VAO activities, as well as AZR activity at the bacterial surface, inside cells, and in the extracellular milieu were all significantly elevated to varying degrees. In the 24–72 h decolorization phase, intracellular Lip and RR activities, extracellular Tyr, VAO, Lip, and AZR activities, and cell surface Tyr, VAO, and RR activities were likewise significantly enhanced at different levels (Figure S6). This spatiotemporal compartmentalization likely facilitates direct attack on the azo bonds of dye molecules by surface-localized reductases during the initial phase, while extracellular oxidases efficiently catalyze subsequent aromatic ring hydroxylation and ring-opening reactions. RB5 exhibits potential toxicity, manifested by the generation of toxic aromatic amines upon cleavage of its azo bonds. 4-Ethenylsulfonylaniline (**5#**, *m*/*z* 184.04268), an aromatic amine compound produced during RB5 degradation, possesses carcinogenicity. This intermediate can be converted by oxidative enzymes into the nontoxic 4-aminobenzenesulfonic acid (**6#**, *m*/*z* 172.00739). Compared to the 24 h sample, the 5 d treated sample no longer contained Compound **5#** (Table S3). Moreover, the extracellular activity of Lip secreted by strain gh32 was significantly elevated at 72 h (Figure S6), indicating a crucial role for Lip in the metabolism of Compound **5#**. 1-Amino-2- naphthol (**18#**, *m*/*z* 158.06114), a naphthylamine derivative generated during RB5 degradation, is potentially carcinogenic. In its metabolic pathway, tyrosinase (Tyr), veratryl alcohol oxidase (VAO), and lignin peroxidase (Lip) collectively act to detoxify this intermediate. Biotoxicity experiments further confirmed that strain gh32 effectively eliminated the toxicity of RB5, and *E. coli* was able to utilize RB5 for growth (Figure 7), indicating that *E. coli* possesses a unique enzymatic system capable of decomposing RB5 into utilizable nutrients.

Under DBG induction, the intracellular activities of laccase (Lac) (Figure S7a), veratryl alcohol oxidase (VAO) (Figure S7b), lignin peroxidase (Lip) (Figure S7c), tyrosinase (Tyr) (Figure S7d), and azoreductase (AZR) (Figure S7e) in strain HL7 were significantly enhanced to varying degrees. At the cell surface, the activities of redox enzymes, including NADH: quinone oxidoreductase 1 (NQO1) (Figure S7f), were all highly significantly enhanced, except for Lip, which showed a significant increase (Figure S7c). Extracellularly, the activities of Lac and Lip also increased significantly (Figure S7a,c). These changes in enzyme activity closely corresponded with the decline in DBG concentration and the accumulation of small molecular products, indicating that they are key enzymes catalyzing the entire process from azo bond cleavage to aromatic ring-opening of DBG.

DBG exhibits genotoxicity. Its degradation product 2-naphthylamine (**7#**, *m*/*z* 144.08078) is a potent carcinogen associated with bladder cancer. It can be converted by laccase (Lac) into the less toxic 1-amino-2-naphthol (**8#**, *m*/*z* 160.07569). Subsequently, Compound **8#** can be detoxified through the action of tyrosinase (Tyr) or veratryl alcohol oxidase (VAO).

DBG undergoes azo bond cleavage mediated by azoreductase (AZR), generating p-phenylenediamine (**2#**, *m*/*z* 109.07602), which is a strong sensitizer with potential carcinogenicity. Compound **2#** is subsequently converted by laccase (Lac) into the toxic aniline (**5#**, *m*/*z* 94.06513), which can cause hematotoxicity and is carcinogenic. Aniline is then detoxified by lignin peroxidase (Lip), yielding 2,7,8-triaminonaphthalen-1-ol (**6#**, *m*/*z* 190.09749). The 7 d treated sample no longer contained Compound **7#** (Table S5). Under DBG induction, the activities of Lac, including intracellular, cell surface, and secreted extracellular, were all significantly increased in strain HL7. Concurrently, the activities of both VAO and Tyr inside the cells and at the cell surface were also significantly elevated (Figure S7).

Under DB15 induction in strain X64, the activities of both Lac (Figure S8a) and azoreductase (AZR) (Figure S8e) were significantly enhanced to varying degrees, indicating the core role of AZR as the “initiating switch” and the responsive induction of Lac by DB15. The activities of RR (Figure S8f) were significantly increased intracellularly and at the cell surface, while Lip activity was significantly enhanced within the cells (Figure S8c). The activities of VAO (Figure S8b) and Tyr (Figure S8d) were also significantly elevated extracellularly and at the cell surface. These increases in redox enzyme activities demonstrate that the strain can not only process the dye intracellularly and at the cell surface but also actively secrete enzymes into the environment for extracellular treatment.

Under sodium citrate supplementation, the extracellular and cell-surface activities of Lac (Figure S9a) and RR (Figure S9f) in strain X64 were significantly enhanced to varying degrees, while Tyr activity was highly significantly increased (Figure S9d). The activities of Lip (Figure S9c), AZR (Figure S9e), and VAO (Figure S9b) were also highly significantly elevated in the extracellular, intracellular, and cell-surface compartments, respectively. With yeast extract addition, the extracellular and cell-surface activities of Lip, VAO, and RR were markedly enhanced; the cell-surface activities of Lac, Tyr, and AZR were highly significantly increased; and extracellular Lac activity was significantly raised (Figure S9). The provision of these metabolic substrates notably boosted the activity of redox enzymes involved in DB15 treatment by strain X64, which was further reflected in a significant increase in degradation efficiency (Table 1).

DB15 is a confirmed carcinogen, with animal studies demonstrating its ability to induce tumors in multiple organs. Among its degradation products, 3,3′-dimethoxybenzidine (**3#**, *m*/*z* 245.12845), benzidine (**8#**, *m*/*z* 185.10733), and O-anisidine (**10#**, *m*/*z* 124.07569) are all potent carcinogens. Compound **3#** can be detoxified through the action of Lac, VAO, and Lip. Alternatively, in another degradation pathway, Compound **3#** can be converted into Compounds **8#** and **10#**, which are gradually metabolized to catechol (Compound **17#**, *m*/*z* 109.02950) and subsequently detoxified by Lac and Tyr. In both the 7 d DB15 treated samples and those supplemented with YE, none of Compounds **8#** or **10#** were detected (Table S7). In the 7 d treated samples, the activities of VAO at the cell surface and extracellularly, as well as intracellular Lip activity, were significantly induced in strain X64. Tyr activity at the cell surface was notably increased, and Lac activity across all compartments was significantly enhanced (Figure S8). In samples supplemented with YE, the activities of Lac, Lip, and VAO at both the cell surface and extracellularly in strain X64 were significantly enhanced, and Tyr activity at the cell surface was markedly induced (Figure S9).

Compared to both control groups, the oxidant FeCl_3_ could highly significantly enhance the activities of Lac (Figure S10a) and Tyr (Figure S10d) across all cellular compartments in strain HL7 during IC treatment, as well as the intracellular and extracellular activities of VAO (Figure S10b) and NADH-DCIP reductase (NDR) (Figure S10e), whereas no marked increase was observed for Lip activity (Figure S10c). The selected oxidant FeCl_3_ promoted the elevation of enzyme activities, an effect that was also reflected in a highly significant improvement in the decolorization rate of IC by strain HL7 (Table 1).

#### 2.2.5. Validation of Oxidoreductase Expression by RT-qPCR

**Figure 10:**
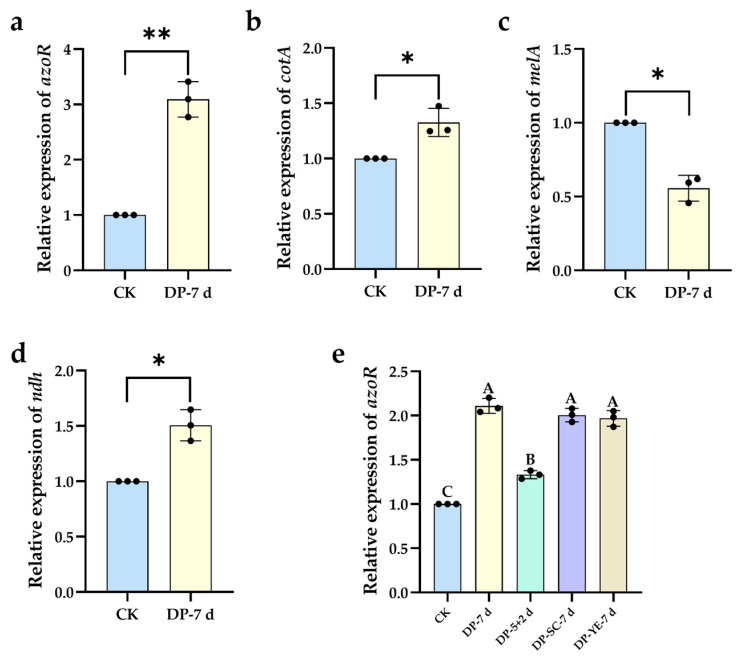
Relative expression of oxidoreductase genes (**a**) *azoR*, (**b**) *cotA*, (**c**) *melA*, and (**d**) *ndh* in *Bacillus* sp. HL7 during decolorization of DBG and (**e**) *azoR* in *Bacillus* sp. X64 during decolorization of DB15. CK: untreated bacterial cells (without dye). DP: degradation product; DP-7 d: DP after 7 d of static treatment with sealing film. DP-5 + 2 d: DP after 5 d of static treatment with sealing film followed by 2 d of agitation; DP-SC-7 d: DP after 7 d of static treatment with sealing film added sodium citrate; DP-YE-7 d: DP after 7 d of static treatment with sealing film added yeast extract. The data of (**a**–**d**) are analyzed by a paired-sample *t*-test using means of three experiments. * indicates significant difference at *p* < 0.05. ** indicates significant difference at *p* < 0.01. The data of (**e**) are analyzed by one-way ANOVA with a Tukey multiple comparisons test using means of three experiments. Different uppercase letters indicate a significant difference at *p* < 0.01.

Analysis of key redox enzyme gene expression via RT-qPCR revealed that strain HL7 exhibited a specific expression profile during DBG decolorization. Throughout the treatment of DBG by strain HL7, the expression levels of *azoR* (Figure 10a), *cotA* (Figure 10b), and *ndh* (Figure 10d) were significantly upregulated to varying degrees, with the exception of *melA* (Figure 10c). In the system where strain X64 decolorized DB15, the expression of *azoR* was highly significantly upregulated across all treatment groups (DP-7 d, DP-5 + 2 d, DP-SC-7 d, and DP-YE-7 d). However, its expression in the group subjected to static incubation for 5 d followed by shaking for 2 d was significantly lower than in the other groups, indicating that oxygen exerts a certain inhibitory effect on the expression of the azoreductase gene. Supplementation with sodium citrate and yeast extract both significantly upregulated the expression of the azoreductase gene, with no significant difference observed between the two substrates (Figure 10e). The observed high degree of correlation between the expression levels of these redox enzyme genes and their corresponding enzyme activities (Figures S7–S9) indicates that strains HL7 and X64 employ transcriptional-level regulation in response to dye stress. These results provide preliminary molecular-level evidence suggesting a correlation between the enhanced enzyme activities observed upon dye exposure and the induced expression of corresponding genes in strains HL7 and X64. The data support the possible involvement of azoreductase and related redox enzymes in the degradation process of the tested azo dyes.

### 2.3. Molecular Docking Analysis Between Enzymes and Dyes

#### 2.3.1. Primary Structure Analysis

The amino acid quantity, molecular weight, theoretical pI, instability index, aliphatic index and grand average of hydropathicity of the protein were analyzed through the ExPASy website. The results are shown in Table 2.

#### 2.3.2. Secondary Structure Analysis

The laccase (Q812W6) and tyrosinase (B7JU04) from *B. cereus* HL7, the laccase from *B. safensis* X64 (A5A677), and the tyrosinase from *R. palustris* gh32 (Q2IY37) exhibited relatively high proportions of random coils, accompanied by lower combined contents of α-helices and β-sheets. This structural feature may be associated with increased conformational flexibility of the amino acid backbone. A higher proportion of random coils can enhance protein flexibility and facilitate conformational transitions, thereby promoting efficient substrate recognition. In contrast, the azoreductases from *B. cereus* HL7 (Q73CJ5), *B. safensis* X64 (O32224), and *R. palustris* gh32 (Q215Z0) displayed higher combined proportions of α-helices and β-sheets, along with lower contents of random coils. This structural composition suggests a more compact and potentially stable protein architecture (Table 3).

#### 2.3.3. Tertiary Structure Analysis

The structural reliability of the predicted three-dimensional protein models was evaluated using the SAVES v6.1 server (https://saves.mbi.ucla.edu (accessed on 30 May 2025)), employing the PROCHECK, Verify3D, and ERRAT tools [27,28,29,30,31]. The ERRAT program is a statistically based method for validating protein structures derived from crystallographic data. All models exhibited excellent overall ERRAT scores. Notably, the azoreductases from strain gh32 (Q215Z0) and strain X64 (O32224), as well as the laccase from strain X64 (A5A677), achieved ERRAT scores exceeding 95, indicating superior structural quality of these protein models. A Verify3D score exceeding 80% indicates that the overall folding architecture of the protein model is reasonable. In the present study, five of the seven enzyme models achieved Verify3D scores above this threshold, whereas the laccase from strain HL7 (Q812W6) and the azoreductase from strain gh32 (Q215Z0) exhibited scores below 80% (Table 4). The relatively low Verify3D scores may be attributed to the presence of flexible loop regions or random coils on the protein surface rather than modeling errors within the catalytic center or the docking pocket. Analysis of the laccase model from strain HL7 using WHATCHECK revealed abnormal side chain conformations for His82, His135, Asn161, and Gln210. However, these residues are located outside the docking site and reside in flexible regions of the protein structure. In the analysis of the azoreductase model from strain gh32, Asp171 exhibited an abnormal side chain planar conformation, while Arg18 and Asp41 showed unusually short interatomic distances. Similarly, these residues are also located outside the docking site. In general, the active region of an enzyme is typically located within internal cavities of the protein structure, whereas local modeling inaccuracies in residues outside the active site are less likely to significantly affect the overall architecture of the catalytic pocket. Therefore, the above results suggest that the active-site region of the constructed model still possesses good structural reliability. PROCHECK analysis further revealed that more than 90% of residues in all seven enzymes were located within the favored, allowed, and generously allowed regions of the Ramachandran plot, indicating conformations consistent with established stereochemical principles. Collectively, these results confirm the satisfactory overall quality of the seven protein models.

#### 2.3.4. Analysis of Connection Results

Molecular docking between enzymes from different bacterial strains and the dyes was performed using AutoDock Vina 1.1.2. The results indicated that hydrogen bonding, hydrophobic interactions, and salt bridges were commonly involved in enzyme–dye binding, with calculated binding energies ranging from −6.7 to −8.7 kcal/mol. Amino acid residues located at the enzyme active sites that contributed to these interactions are highlighted in bold (Table 5).

##### Interaction Between Laccase and Dyes

**Figure 11:**
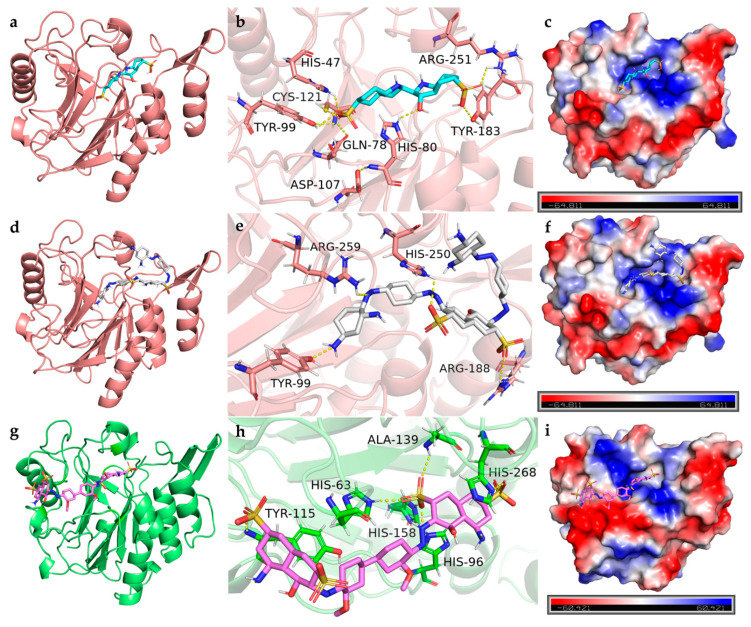
Results of docking between laccase and IC, DBG, and DB15: (**a**–**c**) docking results of *B. cereus* HL7 laccase with IC; (**a**) docking result diagram drawn by PyMOL; (**b**) 3D diagram of hydrogen bond interactions drawn by PyMOL, light pink represents the laccase of strain HL7, cyan represents the IC molecule, red represents the oxygen atom, blue represents the nitrogen atom, and yellow represents the sulfur atom; (**c**) surface electrostatic potential diagram, red represents the negative potential area, and blue represents the positive potential area; (**d**–**f**) docking results of *B. cereus* HL7 laccase with DBG; (**d**) docking result diagram drawn by PyMOL; (**e**) 3D diagram of hydrogen bond interactions drawn by PyMOL, light pink represents the laccase of strain HL7, gray represents the DBG molecule, red represents the oxygen atom, blue represents the nitrogen atom, and yellow represents the sulfur atom; (**f**) surface electrostatic potential diagram, red represents the negative potential area, and blue represents the positive potential area; (**g**–**i**) docking results of *B. safensis* X64 laccase with DB15; (**g**) docking result diagram drawn by PyMOL; (**h**) 3D diagram of hydrogen bond interactions drawn by PyMOL, green represents the laccase of strain X64, violet represents the DB15 molecule, red represents the oxygen atom, blue represents the nitrogen atom, and yellow represents the sulfur atom; (**i**) surface electrostatic potential diagram, red represents the negative potential area, and blue represents the positive potential area.

**Figure 12:**
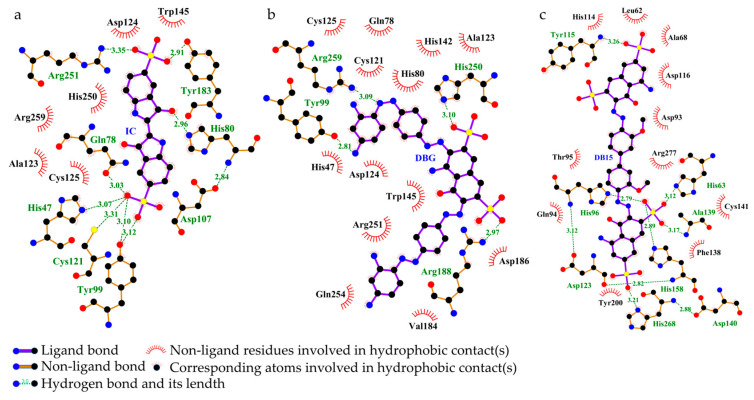
2D interaction diagram drawn by Ligplot^+^: (**a**) interaction between *B. cereus* HL7 laccase and IC; (**b**) interaction between *B. cereus* HL7 laccase and DBG; (**c**) interaction between *B. safensis* X64 laccase and DB15. The blue stick-like framework represents the dye molecules, the green dotted lines represent hydrogen bonds, and the red represents hydrophobic interactions.

The laccase from *B. cereus* HL7 (Q812W6) exhibited binding energies of −7.8 kcal/mol with IC and −7.2 kcal/mol with DBG, indicating a higher binding affinity for IC compared to DBG (Table 5). The docking complexes of HL7 laccase with IC and DBG were visualized using PyMOL (Figure 11a,d). HL7 laccase formed eight and four hydrogen bonds with IC and DBG, respectively; except for the hydrogen bond involving Arg188, all other hydrogen bonds were located within the enzyme active site (Table 5). The laccase primarily formed hydrogen bonds with the SO_3_^−^ functional groups of the IC molecule (Figure 11b). In the docking complex with DBG, Arg259 of HL7 laccase established a hydrogen bond with the azo nitrogen of DBG, indicating that laccase may facilitate cleavage of the azo bond in azo dyes. Arg188 and His250 formed hydrogen bonds with the SO_3_^−^ group of DBG, suggesting that laccase may participate in the desulfonation process (Figure 11e). Furthermore, the residues His47 and His80, which participate in hydrogen bonding between laccase and IC, also contribute to salt bridge formation, while Arg188 and His250, involved in hydrogen bonding with DBG, similarly form salt bridges (Table 5). This suggests that these surfaces are enriched with positively charged basic amino acid residues, such as histidine (bearing imidazolium groups) and arginine (bearing guanidinium groups) (Figure 11c,f). The negatively charged functional groups of IC and DBG, such as SO_3_^−^, are spatially oriented toward the positively charged basic residues of the laccase. This close proximity of oppositely charged groups facilitates the formation of salt bridges (ionic interactions). Notably, His47, His80, and His142, which participate in salt bridge formation between HL7 laccase and IC, as well as His250, which contributes to salt bridge interactions with DBG, are all located within the enzyme active site (Table 5). In addition, IC and DBG molecules engage in hydrophobic interactions with residues such as Ala123 and Val184, as well as aromatic residues like Trp145 (Figure 12a,b).

In comparison, the laccase from *B. safensis* X64 (A5A677) exhibited the lowest binding energy with DB15 (−8.7 kcal/mol), indicating the strongest binding affinity among the enzymes studied (Table 5). The docking complex is shown in Figure 11g. All six hydrogen bonds formed between X64 laccase and DB15 are located within the enzyme active site. The electrostatic potential surface of the enzyme is predominantly blue, indicating a net positive charge (Figure 11h). Positively charged basic residues within the active site, including His96, His158, His268, and Arg277, form four salt bridges with the negatively charged SO_3_^−^ groups of DB15 (Figure 11i, Table 5). The DB15 molecule also engages in hydrophobic interactions with multiple amino acid residues within the enzyme active site. Key residues, including Leu62, Phe138, and Tyr200, are arranged around the aromatic ring framework of DB15, creating a relatively enclosed hydrophobic microenvironment that facilitates stable embedding of the dye ligand within the active site (Figure 12c).

##### Interaction Between Tyrosinase and Dyes

**Figure 13:**
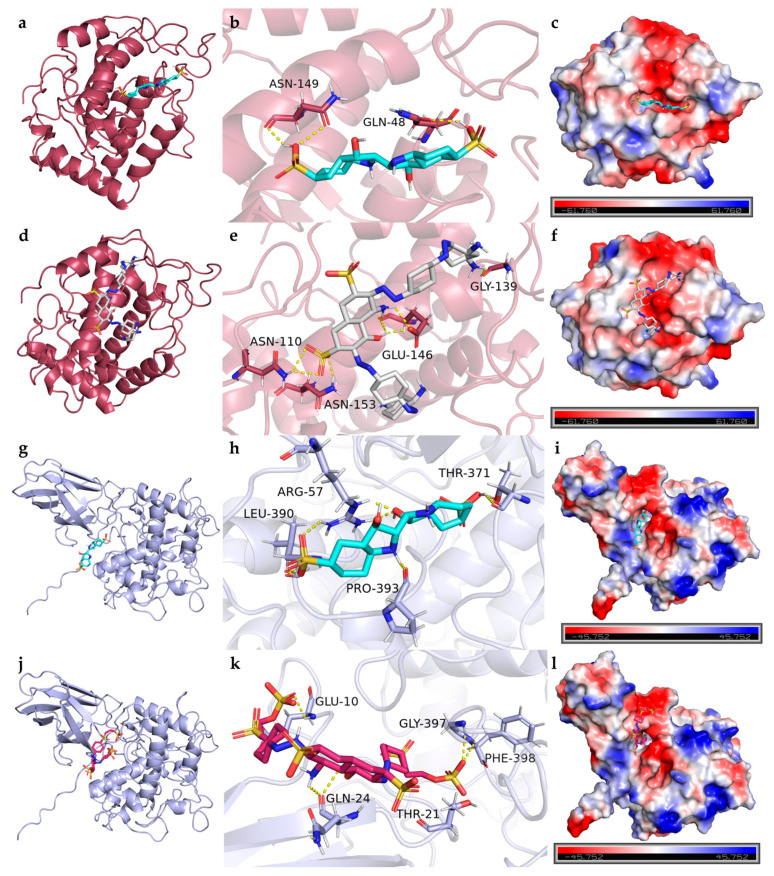
Results of docking between tyrosinase and IC, DBG, RB5: (**a**–**c**) docking results of *B. cereus* HL7 tyrosinase with IC; (**a**) docking result diagram drawn by PyMOL; (**b**) 3D diagram of hydrogen bond interactions drawn by PyMOL, with raspberry red representing the tyrosinase of strain HL7, cyan representing the IC molecule, red representing the oxygen atom, blue representing the nitrogen atom, and yellow representing the sulfur atom; (**c**) surface electrostatic potential diagram, with red representing the negative potential area and blue representing the positive potential area; (**d**–**f**) docking results of *B. cereus* HL7 tyrosinase with DBG; (**d**) docking result diagram drawn by PyMOL; (**e**) 3D diagram of hydrogen bond interactions drawn by PyMOL, with raspberry red representing the tyrosinase of strain HL7, gray representing the DBG molecule, red representing the oxygen atom, blue representing the nitrogen atom, and yellow representing the sulfur atom; (**f**) surface electrostatic potential diagram, with red representing the negative potential area and blue representing the positive potential area; (**g**–**i**) docking results of *R. palustris* gh32 with IC; (**g**) docking result diagram drawn by PyMOL; (**h**) 3D diagram of hydrogen bond interactions drawn by PyMOL, with blue-gray representing the tyrosinase of strain gh32, cyan representing the IC molecule, red representing the oxygen atom, blue representing the nitrogen atom, and yellow representing the sulfur atom; (**i**) surface electrostatic potential diagram, with red representing the negative potential area and blue representing the positive potential area; (**j**–**l**) docking results of *R. palustris* gh32 tyrosinase with RB5; (**j**) docking result diagram drawn by PyMOL; (**k**) 3D diagram of hydrogen bond interactions drawn by PyMOL, with blue-gray representing the tyrosinase of strain gh32 and warm red representing the RB5 molecule; (**l**) surface electrostatic potential diagram, with red representing the negative potential area and blue representing the positive potential area.

**Figure 14:**
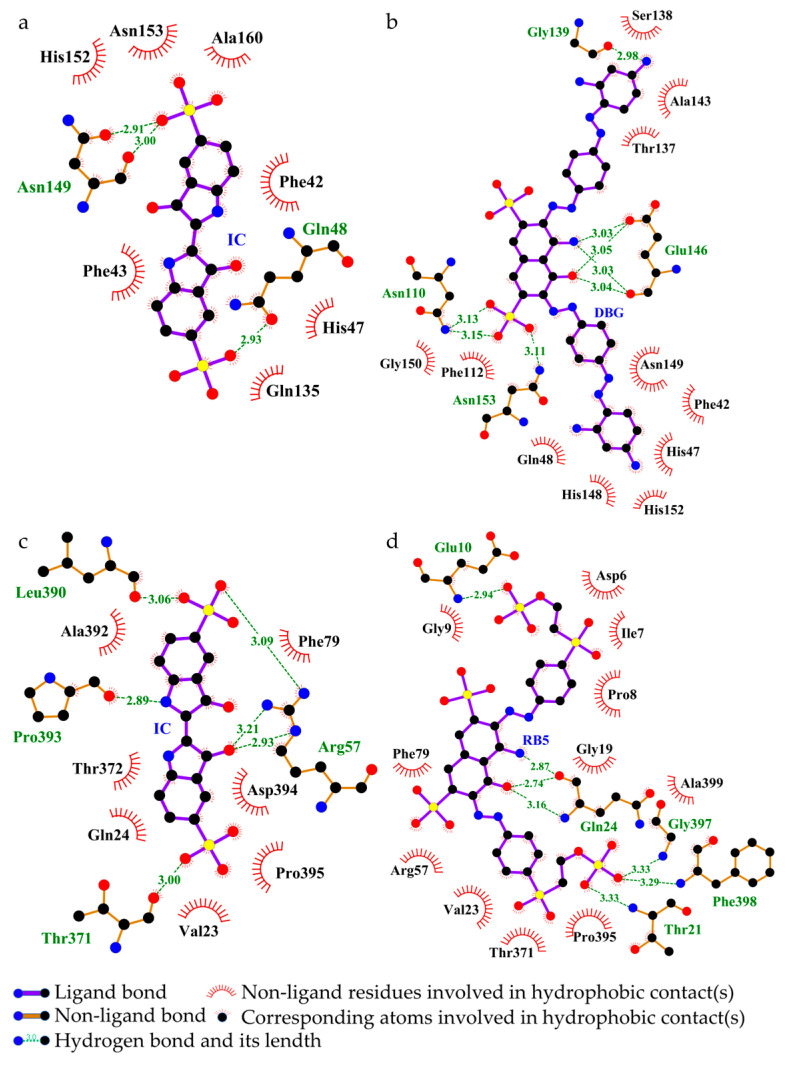
2D interaction diagrams drawn by Ligplot^+^: (**a**) interaction between *B. cereus* HL7 tyrosinase and IC; (**b**) interaction between *B. cereus* HL7 tyrosinase and DBG; (**c**) interaction between *R. palustris* gh32 and IC; (**d**) interaction between *R. palustris* gh32 and RB5. Blue rods represent the dye molecule backbone, yellow rods represent the amino acid backbone, green dotted lines represent hydrogen bonds, red fan-shaped structures represent hydrophobic interactions, red circular shapes represent oxygen atoms, black circular shapes represent carbon atoms, blue circular shapes represent nitrogen atoms, and yellow circular shapes represent sulfur atoms.

The tyrosinase from *B. cereus* HL7 (B7JU04) exhibited a docking binding energy of −6.7 kcal/mol with IC, indicating a relatively limited binding affinity. In contrast, HL7 tyrosinase displayed a stronger binding affinity with DBG, with a binding energy of −7.5 kcal/mol (Table 5). The docking complexes of HL7 tyrosinase with IC and DBG are shown in Figure 13a and Figure 13d, respectively. Within the enzyme active site, residues Gln148 and Asn149 formed one and two hydrogen bonds with the SO_3_^−^ groups of the IC molecule, respectively (Figure 13b). The residue Glu146 of HL7 tyrosinase formed two hydrogen bonds with the -OH and -NH_2_ groups on the naphthalene ring of DBG (Figure 13e). Molecular docking results of HL7 tyrosinase with IC and DBG indicate that both ligands bind within the enzyme active site. The electrostatic potential surface at this site is predominantly red, indicating a net negative charge and suggesting an enrichment of negatively charged acidic residues, such as Asn149 and Glu48 (Figure 13c,f). The negatively charged functional groups of the dye molecules, such as SO_3_^−^, experience electrostatic repulsion from the negatively charged residues in the enzyme active site, which explains why HL7 tyrosinase does not form salt bridge interactions with IC or DBG. Hydrophobic interactions between tyrosinase and the dye molecules are primarily mediated by canonical hydrophobic residues and are further reinforced by the cooperative contribution of hydrophilic residues such as Gln48, Asn153, and Thr137. These hydrophilic residues may indirectly contribute to the formation of a more intricate composite hydrophobic microenvironment that encapsulates the dye ligand through the precise spatial arrangement of their hydrophobic surfaces or carbon backbones (Figure 14a,b). This hydrophobic encapsulation effect enhances the overall stability of the enzyme-dye complex.

The tyrosinase from *R. palustris* gh32 (Q2IY37) exhibited a docking binding energy of −7.6 kcal/mol with RB5, which is lower than that with IC (−7.2 kcal/mol) (Table 5), indicating a stronger binding affinity for RB5. The docking complexes of gh32 tyrosinase with IC and RB5 are shown in Figure 13g and Figure 13j, respectively. In the gh32 tyrosinase-IC complex, Arg57 formed two hydrogen bonds with the -OH group of IC, while Arg57, Thr371, and Leu390 each formed one hydrogen bond with the SO_3_^−^ groups of IC. Additionally, Pro393 established a hydrogen bond with the -NH_2_ group on the IC molecular backbone (Figure 13h). In the gh32 tyrosinase-RB5 complex, residues Glu10, Thr21, Gly397, and Phe398 collectively formed four hydrogen bonds with the SO_3_^−^ groups of RB5, while Gln24 formed one hydrogen bond with the -OH group and two hydrogen bonds with the -NH_2_ group on the naphthalene ring of RB5 (Figure 13k). The electrostatic potential surfaces of the gh32 tyrosinase active site in complexes with IC and RB5 are predominantly red, indicating an abundance of negatively charged acidic residues. Arg57 forms salt bridges with both IC and RB5, suggesting that this residue may play a crucial role in enhancing the stability of the enzyme–dye complexes (Figure 13i,l). The gh32 tyrosinase appears to achieve effective encapsulation of IC and RB5 through a cooperatively formed hydrophobic core composed of diverse amino acid residues. Aliphatic residues such as Val23, Thr372, and Ile7, together with the aromatic residue Phe79, may play a dominant role in stabilizing the conformation of the dye ligands. In addition, certain polar residues, including Gln34, Asp394, and Thr371, may further reinforce the structural stability of the complexes through potential noncanonical hydrophobic contacts (Figure 14c,d).

##### Interaction Between Azoreductase and Dyes

**Figure 15:**
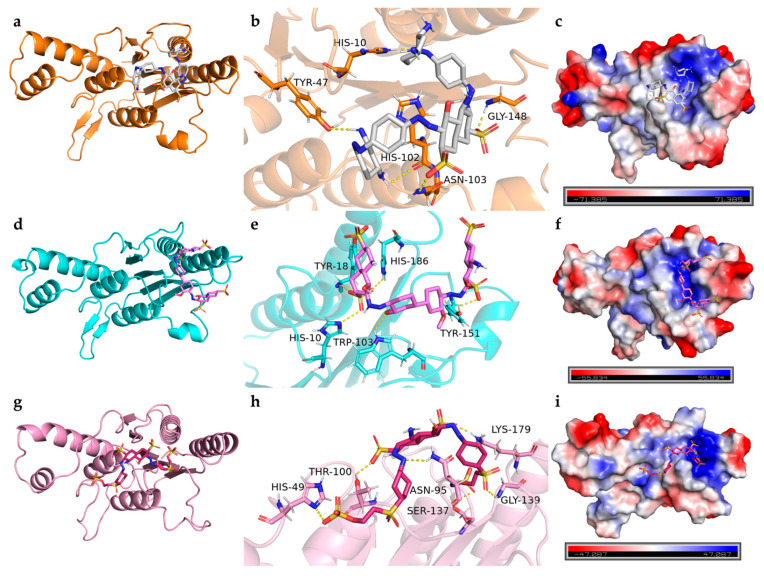
Docking results of azo reductase with DBG, DB15, and RB5: (**a**–**c**) docking results of azo reductase from *B. cereus* HL7 with DBG; (**a**) diagram of docking results drawn by PyMOL; (**b**) 3D diagram of hydrogen bond interactions drawn by PyMOL, orange represents the azo reductase from strain HL7, gray represents the DBG molecule, red represents the oxygen atom, blue represents the nitrogen atom, and yellow represents the sulfur atom; (**c**) surface electrostatic potential diagram, red represents the negative potential area, and blue represents the positive potential area; (**d**–**f**) docking results of azo reductase from *B. safensis* X64 with DB15; (**d**) diagram of docking results drawn by PyMOL; (**e**) 3D diagram of hydrogen bond interactions drawn by PyMOL, cyan represents the azo reductase from strain X64, violet represents the DB15 molecule, red represents the oxygen atom, blue represents the nitrogen atom, and yellow represents the sulfur atom; (**f**) surface electrostatic potential diagram, red represents the negative potential area, and blue represents the positive potential area; (**g**–**i**) docking results of azo reductase from *R. palustris* gh32 with RB5; (**g**) diagram of docking results drawn by PyMOL; (**h**) 3D diagram of hydrogen bond interactions drawn by PyMOL, pink represents the azo reductase from strain gh32, warm red represents the RB5 molecule, red represents the oxygen atom, blue represents the nitrogen atom, and yellow represents the sulfur atom; (**i**) surface electrostatic potential diagram, red represents the negative potential area, and blue represents the positive potential area.

**Figure 16:**
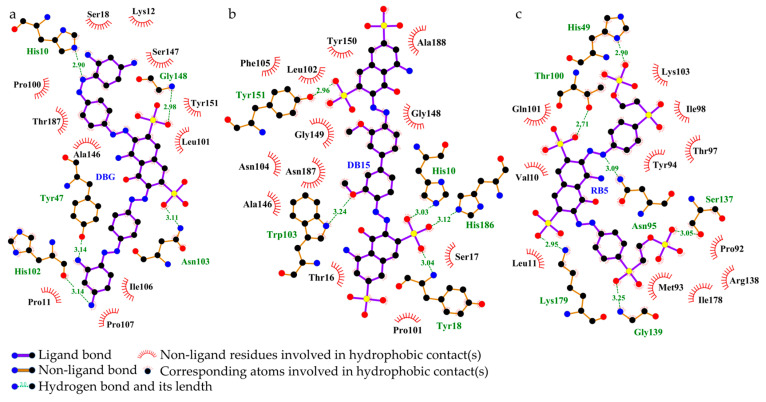
2D interaction diagram drawn by Ligplot^+^: (**a**) interaction between the azoreductase of *B. cereus* HL7 and DBG; (**b**) interaction between the azoreductase of *B. safensis* X64 and DB15; (**c**) interaction between the azoreductase reductase of *R. palustris* gh32 and RB5. The blue rods represent the dye molecule skeleton, the yellow rods represent the amino acid skeleton, the green dotted lines represent hydrogen bonds, the red fan-shaped structures represent hydrophobic interactions, the red circular shapes represent oxygen atoms, the black circular shapes represent carbon atoms, the blue circular shapes represent nitrogen atoms, and the yellow circular shapes represent sulfur atoms.

The azoreductase from *B. cereus* HL7 (Q73CJ5) exhibited a docking binding energy of −7.7 kcal/mol with DBG (Table 5), and the docking complex is shown in Figure 15a. A total of five hydrogen bonds were formed between HL7 azoreductase and DBG, four of which are located within the enzyme active site (Table 5). Within the active site, His10 and His102 formed hydrogen bonds with the -NH_2_ groups of DBG, while Asn103 and Gly148 each formed a hydrogen bond with the SO_3_^−^ groups (Figure 15b). The electrostatic potential surface of the active site region is predominantly blue, indicating a net positive charge (Figure 15c). No salt bridge formation was observed in this complex (Table 5). The DBG molecule is primarily embedded within a hydrophobic region formed by Pro11, Pro100, and aliphatic residues such as Ala146, Ser18, and Leu101, creating a relatively compact hydrophobic framework. This structural arrangement may facilitate the stabilization of the dye within the binding site (Table 5, Figure 16a). The azoreductase from *Bacillus safensis* X64 (O32224) exhibited the lowest binding energy with DB15 among the azoreductases, at −7.9 kcal/mol (Table 5). The docking complex of X64 azoreductase with DB15 is shown in Figure 15d, in which five hydrogen bonds are formed, all within the enzyme active site. Notably, Tyr18 and Tyr151 form hydrogen bonds with the SO_3_^−^ groups of DB15, while Trp103 interacts with the keto group of DB15 via a hydrogen bond (Figure 15e). The electrostatic potential surface indicates that the region of interaction between Tyr and DB15 is predominantly blue (Figure 15f). Within the active site, the positively charged His10 not only forms a salt bridge with the SO_3_^−^ group of DB15 but also participates in hydrogen bonding. Molecular docking analysis revealed that the active site adopts a configuration combining a “hydrophobic core” with “flexible nodes.” The hydrophobic scaffold is primarily composed of residues such as Leu102 and Ala146, while small residues Gly148 and Gly149 are interspersed within this framework Figure 16a). This hybrid arrangement maintains the overall hydrophobic character of the active site while introducing critical local conformational flexibility.

The azoreductase from *R*. *palustris* gh32 (Q215Z0) exhibited a docking binding energy of −7.1 kcal/mol with RB5 (Table 5). The docking complex is shown in Figure 15g. In this complex, the sulfonate groups of RB5 formed five hydrogen bonds with His49, Thr100, Ser137, Gly139, and Lys179 of gh32 azoreductase, while Asn95 formed a hydrogen bond with the azo nitrogen of RB5. These interactions provide molecular docking-level evidence supporting the ability of the azoreductase to cleave the azo bond (Figure 15h). The electrostatic potential surface indicates that the binding region of gh32 azoreductase with RB5 is predominantly blue, corresponding to a positively charged region (Figure 15i). Positively charged residues involved in hydrogen bonding, such as His49 and Lys179, also participate in salt bridge formation, resulting in two salt bridges in total (Table 5). Within the binding site, hydrophobic residues, including Leu11, Met93, Ile98, Ile178, and Tyr94 are arranged longitudinally, forming a linear hydrophobic channel that guides the dye ligand into the active site. The shape of this channel is highly complementary to the aromatic backbone of the RB5 molecule, allowing it to be effectively accommodated and directionally guided within the bulky hydrophobic structure of the dye. This hydrophobic complementarity may provide a key driving force for the fitting and anchoring of RB5 within the binding site of strain gh32 azoreductase, thereby significantly enhancing the overall stability of the enzyme–dye complex (Figure 16c).

## 3. Discussion

This study systematically investigated the degradation efficiency, pathways, and detoxification effects of three bacterial strains (*R. palustris* gh32, *B. cereus* HL7, and *B. safensis* X64) on indigoid and azo dyes. A multi-level analysis was conducted, integrating enzymology, gene expression, and molecular docking techniques to elucidate the process from macro-scale phenomena to micro-scale mechanisms. The findings not only confirm the great potential of microbial dye degradation but also, through comparison with existing literature, shed light on both unique findings and general principles, thereby offering novel insights for developing efficient bioremediation strategies.

### 3.1. Multi-Enzyme Collaborative Catalysis Degradation Pathways: From Azo Bond Cleavage to Aromatic Ring Cleavage

This study reveals that bacteria employ a sequential three-stage enzymatic strategy to degrade complex dye molecules, which critically relies on the precise temporal and spatial orchestration of multiple oxidoreductases. First, azoreductase (AZR) acts as the “initiation switch” for degradation, responsible for the reductive cleavage of the core chromophoric structure of the dye molecule, specifically the azo bond (-N=N-) (Figure 3, Figure 4 and Figure 5), as exemplified by the degradation of DB15 yielding 3,3′-dimethoxybenzidine (**3#**, *m*/*z* 245.12845). Simultaneously, RT-qPCR results in this study revealed that the *azoR* gene was significantly upregulated under dye stress (Figure 10a,e). Furthermore, molecular docking elucidated specific interactions between key amino acid residues in the AZR active site—such as His 10 (Table 5, Figure 15f), Lys 179, and His 49 (Table 5, Figure 15i)—and the sulfonic acid groups as well as the nitrogen atoms of the azo bonds in the dye molecules. These findings provide direct molecular evidence for the substrate recognition and catalytic mechanism of AZR. Collectively, the above findings confirm the central role of azoreductase (AZR) in the degradation of azo dyes. This enzyme initiates the reductive cleavage of azo bonds in textile wastewater by utilizing NADH as an electron donor, facilitating the primary degradation of these dyes [32,33]. For instance, during the degradation of methyl red by *Bacillus* sp. strain UN2, AZR activity was significantly enhanced and showed a positive correlation with the decolorization efficiency [34]. Subsequently, oxidative enzyme systems represented by laccase (Lac), lignin peroxidase (LiP), veratryl alcohol oxidase (VAO), and tyrosinase (Tyr) were significantly induced (Figures S6–S9). These enzymes are responsible for the further transformation of primary aromatic amine products through processes such as hydroxylation and oxidative ring-opening. This multi-enzyme collaborative model termed ”reductive initiation followed by oxidative deepening”. The cooperative induction and expression of these oxidative enzymes indicate their significant role in the dye degradation process, which is consistent with the multi-enzyme induction and expression observed during the degradation of the azo dye Remazol Orange 3R by the combined plant system AG, composed of Aster amellus Linn. and Glandularia pulchella (Sweet) Tronc [35].

Further subcellular enzyme activity analysis revealed a distinct spatial compartmentalization of enzyme localization: reductases (AZR and RR) are primarily localized at the cell surface or within the cytoplasm, facilitating direct contact and reduction of the dyes. In contrast, oxidative enzymes (Lip, Lac, and VAO) are largely secreted extracellularly, where they efficiently catalyze subsequent aromatic ring oxidation and cleavage reactions. This spatial division of labor likely represents an adaptive strategy employed by bacteria, compensating for their lack of the extensive extracellular enzyme secretion systems characteristic of fungi.

In summary, this study clearly delineates the bacterial degradation pathway characterized by an “AZR-mediated reductive initiation to oxidative enzyme-driven deep conversion” sequence, spatially coordinated through” intracellular/surface reduction coupled with extracellular oxidation”. This provides a systematic framework for understanding the molecular mechanisms underlying efficient bacterial degradation of azo dyes. Integrated with dynamic enzyme activity analysis, the work constructs a degradation pathway map that is more detailed and comprehensive than those presented in prior studies.

### 3.2. The Intrinsic Unity of Decolorization and Detoxification: Structure-Dependent Toxicity

A key conclusion of this study is that thorough detoxification is founded upon the fundamental disruption of the dye molecular structure, rather than merely decolorization [36,37]. The disappearance or blue shift of characteristic dye peaks in UV-Vis spectra only indicates the destruction of the chromophoric conjugated system; genuine toxicity elimination requires the further transformation of toxic intermediates, as confirmed by UPLC-Orbitrap-HRMS. For example, the highly toxic nitrobenzene (**8#**, *m*/*z* 124.03931) generated during IC degradation was converted by tyrosinase (Tyr) into the less toxic 2 amino 3,4,5 trihydroxybenzoic acid (**12#**, *m*/*z* 186.03970) and finally underwent ring-opening to form the aliphatic intermediate (1 aminocyclohexyl)methanol (**13#**, *m*/*z* 130.12264) (Figure 2). This aligns with conclusions from previous studies, indicating that the decolorization of dyes does not always equate to a reduction in toxicity, as certain degradation intermediates may exhibit higher toxicity than the parent dye compounds [38,39]. This study confirms the low toxicity of the degradation products through a multi-level biological toxicity assessment (involving microorganisms, plants, and erythrocytes), which is consistent with the structural disruption revealed by FTIR and UPLC-Orbitrap-HRMS analyses, thereby establishing a complete evidence chain from chemical structural changes to biological effects.

### 3.3. Interaction and Binding Mode Analysis Between the Enzyme and Dye

Molecular docking has provided an effective approach for investigating the interactions between enzymes and dyes and has gradually become an important tool in studies of enzymatic dye degradation [19]. The docking results indicated that the binding energies of all enzyme–dye complexes ranged from −6.7 to −8.7 kcal/mol, suggesting that each dye molecule could adopt a relatively stable binding conformation within the active pocket of the enzyme. Further analysis revealed that hydrogen bonds, hydrophobic interactions, and salt bridges played crucial roles in maintaining the stability of the complexes.

Specifically, polar functional groups in dye molecules, such as sulfonate (SO_3_^−^), hydroxyl (−OH), and amino (−NH_2_) groups, frequently act as hydrogen bond donors or acceptors. These groups can form hydrogen bonding networks with polar amino acid residues at the active site, including His, Arg, Asn, and Gln, thereby enhancing the binding stability of the substrate within the catalytic pocket. Meanwhile, hydrophobic amino acid residues surrounding the active site can form a hydrophobic microenvironment around the aromatic ring structures of the dye molecules. This environment may further stabilize the enzyme–substrate complex through a hydrophobic enclosure effect (Table 5).

In the laccase system, the active sites of laccases from strains HL7 and X64 are enriched with positively charged amino acid residues. For example, histidine residues can form salt bridges or hydrogen bond interactions with the negatively charged sulfonate groups of dye molecules. This charge complementarity facilitates the oriented binding of dye molecules within the active site and enhances the overall stability of the enzyme–substrate complex. In addition, multiple hydrophobic residues interact with the aromatic backbone of the dye molecules, creating a relatively stable hydrophobic environment that favors the binding and positioning of the dye molecules [40].

In the tyrosinase system, the tyrosinases from strains HL7 and gh32 were able to form multiple hydrogen bonds with dye molecules within the active site region. However, the surface electrostatic potential of these enzymes was predominantly negative, which makes it difficult to form salt bridges with the negatively charged sulfonate groups of the dyes. This suggests that, in this system, hydrogen bonding and hydrophobic interactions may serve as the primary driving forces for maintaining the stability of the enzyme–dye complexes.

For the azoreductase system, the active sites of azoreductases from strains HL7, X64, and gh32 were enriched with positively charged amino acid residues and were surrounded by several hydrophobic residues. Notably, in the docking results of the HL7 azoreductase with DBG and the gh32 azoreductase with RB5, hydrogen-bond interactions were observed between the enzyme and the azo bond of the dye molecules. This finding suggests that azo dyes may initially undergo azo bond cleavage catalyzed by azoreductase, followed by further degradation through the synergistic action of other degradative enzymes [41]. These results provide structural evidence at the molecular level supporting the involvement of azoreductase in the cleavage of azo bonds.

It should be emphasized that, due to differences in structural composition and active-site conformations among enzymes derived from different strains, the molecular docking results obtained in this study were primarily used to analyze the potential binding modes and molecular interaction characteristics between the enzymes and dye molecules. The purpose of this analysis was to provide structural insights into the possible roles of these enzymes in dye degradation. Therefore, these docking results were not intended to be used for direct comparisons of degradation efficiency among different strains.

### 3.4. Strain-Specific Degradation Characteristics and Performance Enhancement Strategies

This study revealed that while all three bacterial strains were capable of degrading dyes, they exhibited distinct substrate preferences: strain gh32 demonstrated high proficiency in treating IC and RB5, strain HL7 was most efficient toward DBG, and strain X64 specialized in degrading DB15. This functional differentiation is associated with their unique enzymatic compositions and regulatory mechanisms, suggesting that functional complementarity among strains could be considered when constructing microbial consortia for treating complex dye wastewater [42].

Furthermore, this study demonstrated that the degradation performance could be effectively enhanced by exogenous additives. The addition of Fe^3+^ likely serves as a cofactor for oxidative enzymes (e.g., Lac) or participates in Fenton-like reactions to facilitate oxidation [20], significantly increasing both the decolorization rate of IC by strain HL7 and the corresponding enzyme activity. The addition of sodium citrate or yeast extract, on the one hand, functioned as readily utilizable carbon sources that enhanced microbial metabolism and enzyme synthesis (e.g., significantly upregulating the expression of the *azoR* gene in strain X64); on the other hand, they could act as redox mediators or stabilizers, thereby improving the activity and stability of extracellular enzymes. This aligns with the widely adopted strategy of using cometabolic substrates to enhance dye degradation efficiency [12]. However, the present study not only observed efficiency improvement but also elucidated the underlying physiological and molecular regulatory mechanisms through enzyme activity assays and gene expression analysis, thereby providing deeper insights for optimizing the operational parameters of biological treatment processes.

## 4. Materials and Methods

### 4.1. General

Indigo carmine (IC) and Reactive Black 5 (RB5) were obtained from Sigma-Aldrich (Shanghai, China). Direct Black G (DBG) was purchased from Shanghai Linn En Technology Development Co., Ltd. (Shanghai, China), and Direct Blue 15 (DB15) was supplied by Shanghai Macklin Biochemical Co., Ltd. (Shanghai, China). *Escherichia coli* SWDX and *Bacillus subtilis* WD were identified, characterized, and preserved in our laboratory. *Rhodopseudomonas palustris* gh32 (GenBank: MK942701 for 16S rDNA) was isolated from sediment-water samples collected at a depth of 13 m in the Liaohe River estuary, Panjin City, Liaoning Province. *Bacillus cereus* HL7 (GenBank: PP514600 for 16S rDNA) was isolated from soil samples collected in Gaolan County, Gansu Province. *Bacillus safensis* X64 (GenBank: PP514623 for 16S rDNA) was isolated from sediment-water samples collected in Dongxing County, Heilongjiang Province.

### 4.2. Bacterial Decolorization Capability on Azo Dyes and Indigoid Dyes

The bacterial strains were cultured at 37 °C until the logarithmic growth phase and harvested by centrifugation at 4000 rpm. The cell pellets were then washed three times with sterile water to completely remove residual culture medium components. Finally, the cells were resuspended in buffer, and the concentration was adjusted to an OD_600_ of 1.8 to obtain a standardized bacterial suspension for the study.

The decolorization system for IC by strain gh32 was established in Erlenmeyer flasks containing 20% (*v*/*v*) bacterial suspension of *R. palustris* gh32 and 50 mg/L IC, prepared in a citrate-disodium hydrogen phosphate buffer (pH 7.0). The cultures were incubated at 30 °C with shaking at 135 rpm under dark conditions for 6 h. Following incubation, the cultures were centrifuged, and the supernatants were collected for spectrophotometric analysis. Absorbance was measured at 610 nm (OD_610_), and the decolorization efficiency was expressed as the percentage decrease in OD_610_ relative to the initial value. The decolorization system for RB5 by strain gh32 was conducted in Erlenmeyer flasks containing 10% (*v*/*v*) bacterial suspension of *R*. *palustris* gh32 and 150 mg/L RB5 in a citrate-disodium hydrogen phosphate buffer (pH 7.0). The cultures were incubated at 37 °C under static conditions with protection from light for 72 h. After incubation, the supernatants were collected for spectrophotometric analysis. Absorbance was measured at 596 nm (OD_596_), and the decolorization efficiency was calculated as the percentage reduction in OD_596_ relative to the initial value. The decolorization system for DBG by strain HL7 was performed in centrifuge tubes containing 30% (*v*/*v*) bacterial suspension of *Bacillus cereus* HL7 and 50 mg/L DBG in a citrate-disodium hydrogen phosphate buffer (pH 7.0). The tubes were sealed with parafilm and incubated at 37 °C under static, dark conditions for 96 h. Following incubation, the samples were centrifuged, and the supernatants were collected for spectrophotometric analysis. Absorbance was measured at 648 nm (OD_648_), and the decolorization efficiency was expressed as the percentage decrease in OD_648_ relative to the initial value. The decolorization system for DB15 by strain X64 was conducted in centrifuge tubes containing 30% (*v*/*v*) bacterial suspension of *Bacillus safensis* X64 and 100 mg/L DB15. Sodium citrate or yeast extract was supplemented as an additional substrate at a concentration of 5 g/L. The reaction mixtures were prepared in a citrate-disodium hydrogen phosphate buffer (pH 7.0), sealed with parafilm, and incubated at 37 °C under static, dark conditions for 96 h. After incubation, the samples were centrifuged, and the supernatants were collected for spectrophotometric analysis. Absorbance was measured at 594 nm (OD_594_), and the decolorization efficiency was calculated as the percentage decrease in OD_594_ relative to the initial value. A substrate-free system was used as the control. The decolorization system for IC by strain HL7 was established in Erlenmeyer flasks containing 50 mg/L IC. An aliquot (2 mL) of *Bacillus cereus* HL7 culture (OD_600_ = 1.8) pretreated with 0.8 mmol/L FeCl_3_ for 4 d was added to 17.8 mL of citrate-disodium hydrogen phosphate buffer (pH 7.0). The reaction mixtures were incubated at 37 °C with shaking at 135 rpm under dark conditions for 120 h. After incubation, the cultures were centrifuged, and the supernatants were collected for spectrophotometric analysis. Absorbance was measured at 610 nm (OD_610_), and the decolorization efficiency was expressed as the percentage decrease in OD_610_ relative to the initial value. A bacterial suspension without FeCl_3_ pretreatment was used as the control.

### 4.3. Mechanisms of Dye Decolorization by Bacterial Strains

#### 4.3.1. Scanning Spectrophotometric Analysis

After treatment, the samples were centrifuged at 12,000 rpm for 5 min, and the supernatants were carefully collected and subjected to full-wavelength scanning using a multimode microplate reader (Spark, Tecan, Switzerland).

#### 4.3.2. Identification of Degradation Intermediates Using UPLC-Orbitrap-HRMS and Development of Degradation Pathway Diagrams

The supernatant of the sample was filtered and sterilized using a 0.22 μm microporous filter membrane. Untreated parent dye, replacing the sample, served as the control for the identification of metabolites. Then, the sample was extracted using a universal polymer solid-phase extraction column (HLB) to remove impurities. The processed samples were subsequently analyzed by UPLC-Orbitrap-HRMS (mass spectrometer Q Exactive Focus, liquid chromatography Vanquish, Thermo Scientific, Waltham, MA, USA). Chromatographic separation was performed on a column (100 × 2.1 mm, 3 µm particle size) maintained at 30 °C. The mobile phase consisted of (A) acetonitrile containing 0.1% formic acid and (B) water containing 0.1% formic acid. Gradient elution was applied under the following conditions: *t* = 0–12–14–14.1–22 min for IC and RB5, and *t* = 0–1–14–17–17.1–20 min for DBG and DB15. The ESI parameters for the positive and negative modes are as follows: Spray voltage 3.2 kV, Capillary temperature 300.00 °C, Sheath gas flow rate 40.00 Arb, Auxiliary gas flow rate 15.00 Arb, Maximum spray current 100.00 µA, S lens RF level 50.00%. The obtained liquid chromatography-mass spectrometry data was processed and analyzed using Xcalibur 4.1 software.

#### 4.3.3. Detoxification Evaluation of Biodegraded Dye Products

*Escherichia coli* and *Bacillus subtilis* were individually inoculated into the samples and incubated at 37 °C with shaking at 150 rpm for 12 h. To eliminate potential interference from culture medium components in the toxicity test, PBS (pH 7.0) was used as the reaction medium to establish a relatively simplified experimental system. Two control groups were included: (1) PBS (pH 7.0) alone served as the negative control to assess the normal growth status of the test strains in a non-toxic environment (CK); (2) the original dye dissolved in PBS (pH 7.0) served as the positive control to evaluate the toxicity of the untreated dye. Following incubation, the cultures were serially diluted tenfold and spread onto beef extract-peptone agar plates, which were then incubated at 37 °C overnight. Colony-forming units (CFUs) were enumerated using the plate counting method. All experiments were performed in triplicate.

Notably, in the dye degradation experimental system, no culture medium components were added to support microbial growth. As a result, the degrading bacteria remained metabolically inactive and did not proliferate during the process, thereby excluding the possibility that any metabolites toxic to the test strains could have been produced.

Uniform, intact, and plump tobacco and mung bean seeds from the same batch were stored at room temperature. They were surface-sterilized with 75% (*v*/*v*) ethanol for 30 s, followed by disinfection with 9% (*w*/*v*) sodium hypochlorite for 2 min. The seeds were then rinsed thoroughly with distilled water six to seven times. Subsequently, 10 mL of the test sample was added to each Petri dish. Sterile water was used in place of the sample as the negative control group (CK). The original (untreated) dye was used in place of the sample to serve as the dye control group. Fifty seeds were evenly distributed in each dish and incubated in a plant growth chamber under controlled conditions (25 °C and 95% relative humidity). After 7 d of incubation, seed germination rates were calculated. Each experimental group was performed in triplicate. Seeds were considered germinated when the radicle length exceeded half of the seed length.

Peripheral blood was collected from the fingertips of healthy volunteers, and erythrocytes were obtained by washing the samples three times with sterile normal saline. The erythrocytes were then resuspended in normal saline (prepared as a 0.5% suspension) to prepare a red blood cell suspension. An aliquot of 25 μL of the test sample was added to 100 μL of the erythrocyte suspension and incubated at 37 °C under static conditions for 1 h. Following incubation, erythrocyte counting was performed using a hemocytometer. Normal saline was used in place of the sample as the negative control group (CK). The original (untreated) dye was used in place of the sample to serve as the dye control group.

#### 4.3.4. Dye-Induced Changes in Oxidoreductase Activity Within Biological Systems

The preparation of the bacterial suspension and the decolorization treatment procedure were identical to those described in Section 4.2, with the only difference being the incubation time of the strains with the dyes. Specifically, strain gh32 was incubated with IC for 4 h and with RB5 for 24 h and 72 h. Strain HL7 was incubated with DBG for 7 d and with IC for 5 d. Strain X64 was incubated with DB15 for 7 d. Untreated bacterial cells were used as the control (labeled with the corresponding strain names).

The cultures were centrifuged at 7000 rpm for 10 min, and the resulting supernatants were collected for the determination of extracellular enzyme activity. The cell pellets were then harvested by centrifugation at 7000 rpm for 10 min and disrupted using an ultrasonic cell disruptor (SCIENI-IID, Ningbo Scientz Biotechnology Co., Ltd., Ningbo, China) at a power output of 600 W, with alternating cycles of 3 s sonication and 3 s pause for a total duration of 30 min. The resulting cell lysates were subsequently centrifuged at 7000 rpm for 10 min, and the supernatants were collected for the assessment of intracellular enzyme activity.

The cells were harvested by centrifugation at 7000 rpm for 10 min, washed three times with sterile water, and subsequently resuspended in sterile water to the appropriate concentration for the determination of cell surface-associated enzyme activity.

Lignin peroxidase activity assay: The reaction mixture consisted of 0.2 mL of 10 mmol/L resveratrol, 0.02 mL of 20 mmol/L hydrogen peroxide, 0.2 mL of crude enzyme solution, and 0.4 mL of tartrate-potassium sodium tartrate buffer (pH 3.0). The reaction was carried out at 37 °C for 10 min, after which the absorbance was measured at 310 nm (OD_310_) [43]. Veratryl alcohol oxidase activity assay: The reaction mixture comprised 0.3 mL of 10 mmol/L resveratrol, 0.5 mL of crude enzyme solution, and 0.5 mL of citrate-disodium hydrogen phosphate buffer (pH 3.0). The reaction was incubated at 37 °C for 10 min, after which the absorbance was measured at 310 nm (OD_310_) [44]. Tyrosinase activity assay: The reaction mixture consisted of 0.3 mL of 10 mmol/L L-3,4-dihydroxyphenylalanine (L-DOPA), 0.1 mL of crude enzyme solution, and 0.7 mL of citrate-disodium hydrogen phosphate buffer (pH 6.8). The reaction was incubated at 37 °C for 10 min, after which the absorbance was measured at 475 nm (OD_475_). Riboflavin reductase activity assay: The reaction mixture comprised 0.5 mL of 10 μmol/L riboflavin, 0.3 mL of 25 μmol/L NADPH, 0.2 mL of crude enzyme solution, and 0.5 mL of Tris-HCl buffer (pH 7.4). The reaction was carried out at 37 °C for 10 min, and the absorbance was subsequently measured at 340 nm (OD_340_). Azoreductase activity assay: The azoreductase activity assay was conducted with minor modifications based on the method reported by Misal et al. [45]. The reaction mixture consisted of 0.6 mL of 24 μmol/L Orange II, 0.2 mL of 1 mmol/L NADH, 0.5 mL of crude enzyme solution, and 1.0 mL of PBS, pH 7.0. The reaction was incubated at 37 °C for 10 min, after which the absorbance was measured at 482 nm (OD_482_). Laccase activity assay: The reaction mixture comprised 0.25 mL of 1 mmol/L syringaldazine, 0.1 mL of crude enzyme solution, and 1.15 mL of citrate-disodium hydrogen phosphate buffer (pH 7.0). The reaction was carried out at 37 °C for 10 min, and the absorbance was subsequently measured at 525 nm (OD_525_). NADH: quinone oxidoreductase 1 activity assay: The reaction mixture consisted of 0.2 mL of 1 mmol/L NADH, 0.2 mL of 0.04 mmol/L menadione, 0.5 mL of crude enzyme solution, and 1.6 mL of PBS, pH 7.0. The reaction was carried out at room temperature for 2 min, after which the absorbance was measured at 340 nm (OD_340_). NADH-DCIP reductase activity assay: The reaction mixture comprised 0.1 mL of 50 μmol/L NADH, 0.3 mL of 50 μmol/L 2,6-dichlorophenolindophenol sodium salt (DCIP), 0.2 mL of crude enzyme solution, and 2.0 mL of PBS, pH 7.4. The reaction was incubated at 35 °C for 3 min, and the absorbance was subsequently recorded at 590 nm (OD_590_).

#### 4.3.5. Validation of Oxidoreductase Gene Expression by RT-qPCR

To validate the expression of oxidoreductase-related genes in the strains during dye treatment, RT-qPCR was employed to analyze the transcriptional levels of *azoR* (azoreductase gene), *cotA* (laccase gene), *melA* (tyrosinase gene), and *ndh* (NADH: quinone oxidoreductase 1 gene). Conserved mRNA sequences of these genes were retrieved from the NCBI nucleotide database, and gene-specific primer pairs were designed using SnapGene software (version 4.3.6). The 16S rDNA gene was used as the internal reference control in all reactions. All primers were synthesized by Sangon Biotech Co., Ltd. (Shanghai, China), and detailed primer information is provided in Supplementary Table S8.

Untreated bacterial cells were used as the control (CK). The procedures for bacterial suspension preparation and decolorization were identical to those described in Section 4.2, with the treatment duration being the sole variable. Strain gh32 was treated with IC for 10 h and with RB5 for 24 h and 5 d. Strain HL7 was exposed to DBG for 5 and 7 d, and strain X64 was exposed to DB15 for 7 d and for a consecutive period of 5 + 2 d. After the designated treatment periods, the cells were harvested for total RNA extraction.

Total RNA was extracted from the harvested bacterial strains using a Bacteria Total RNA Isolation Kit, and cDNA was synthesized from the total RNA template using the HiFiScript cDNA Synthesis Kit (Cowin Biosciences Co., Ltd., Taizhou, China). Quantitative real-time PCR analysis of mRNA expression was performed using TB Green^®^ Premix Ex Taq™ (Tli RNaseH Plus) (TaKaRa Bio Inc., Shiga, Japan) on a LightCycler 480 II Real-Time PCR System (Roche Diagnostics, Grenzach-Wyhlen, Germany), with three technical replicates for each sample. Each PCR reaction was performed in a total volume of 20 μL, containing TB Green Premix Ex Taq (Tli RNaseH Plus), 70 ng of cDNA template, and 4 pmol of each primer. The thermal cycling program was set as follows: an initial denaturation at 95 °C for 30 s; 50 cycles of denaturation at 95 °C for 5 s, followed by annealing and extension at 60 °C for 30 s. Melting curve analysis was conducted with sequential steps at 95 °C for 5 s, 60 °C for 60 s, and 95 °C, followed by a cooling step at 50 °C for 30 s. Relative gene expression levels were calculated using the 2^−ΔΔCt^ method.

### 4.4. Study on Molecular Docking Between Enzymes and Dyes

#### 4.4.1. Homology Modeling

Homology modeling was employed to construct the three-dimensional structures of key oxidoreductases involved in dye degradation in the respective bacterial strains. The amino acid sequences of the target degradation enzymes were retrieved from the National Center for Biotechnology Information (NCBI) protein database (https://www.ncbi.nlm.nih.gov/protein/ (accessed on 7 May 2025)) [46]. The primary structural properties of the proteins, including number of amino acids, molecular weight, theoretical pI, instability index, aliphatic index, and grand average of hydropathicity (GRAVY), were subsequently analyzed using the ExPASy ProtParam server (https://web.expasy.org/protparam (accessed on 28 May 2025)) [47]. The secondary structures of the proteins were analyzed using the SOPMA server (https://npsa.lyon.inserm.fr/cgi-bin/npsa_automat.pl?page=/NPSA/npsa_sopma.html (accessed on 28 May 2025)), which predicts residues forming α-helices, β-sheets, and random coils [48]. Subsequently, the amino acid sequences were uploaded to the SWISS-MODEL server (https://swissmodel.expasy.org (accessed on 29 May 2025)) for homology modeling [49]. A total of seven degradation enzyme models were constructed using experimentally resolved amino acid sequences and three-dimensional protein structures from the UniProt database as templates. Generally, models with sequence identities greater than 40% are considered suitable for structural analysis. The laccase (Q812W6), tyrosinase (B7JU04), and azoreductase (Q73CJ5) from *Bacillus cereus* HL7 were modeled based on templates exhibiting sequence identities of up to 98.9%, 100%, and 100%, respectively. The laccase (A5A677) and azoreductase (O32224) from *Bacillus safensis* X64 were constructed using templates with sequence identities of up to 55.84% and 100%, respectively. Similarly, the tyrosinase (Q2IY37) and azoreductase (Q215Z0) from *Rhodopseudomonas palustris* gh32 were modeled based on templates with sequence identities of up to 100% and 72.77%. The rationality of the predicted three-dimensional protein structures was evaluated using the WHATCHECK, PROCHECK, Verify3D, and ERRAT tools available on the SAVES v6.1 server (https://saves.mbi.ucla.edu (accessed on 30 May 2025)) [27,28,29,30,31]. The three-dimensional structures of the dye molecules were obtained from the PubChem database (https://pubchem.ncbi.nlm.nih.gov/ (accessed on 30 May 2025)) [50]. All dye ligands were subjected to energy minimization using the MM2 force field implemented in Chem3D prior to subsequent analyses.

#### 4.4.2. Molecular Docking

The ProteinPlus server (https://proteins.plus/ (accessed on 15 November 2025)) was employed to predict protein active-site locations in order to identify potential binding regions between the enzymes and dye molecules, and the highest-scoring active site was selected as the docking site [51]. The predicted active site drug scores varied among the enzymes from different strains. In strain HL7, the drug scores of laccase, tyrosinase, and azoreductase were 0.78, 0.76, and 0.55, respectively. For strain X64, the drug scores of laccase and azoreductase were 0.74 and 0.60, respectively. In strain gh32, the drug scores of tyrosinase and azoreductase were 0.78 and 0.65, respectively. Molecular docking was performed using AutoDock Vina 1.1.2 [52,53], in which the Lamarckian genetic algorithm was applied to calculate the binding free energies of flexible ligands interacting with rigid protein structures. The optimal docking conformations were selected based on the lowest binding energy. The interactions between dye molecules and protein active sites, as well as their interactions with key amino acid residues, were further analyzed and visualized using PyMOL 2.6, LigPlot^+^ 2.3, and the PLIP server (https://plip-tool.biotec.tu-dresden.de/plip-web/plip/index (accessed on 22 November 2025)) [54,55,56].

### 4.5. Statistical Analysis of Experimental Data

All experiments were conducted with three biological replicates, and the results are expressed as mean ± standard deviation. Statistical analysis was performed using one-way ANOVA followed by Tukey’s test in SPSS 17.0. Differences were considered statistically significant at *p* < 0.05 (indicated by different lowercase letters) and highly significant at *p* < 0.01 (indicated by different uppercase letters). To compare the differences in a variable for the same set of samples before and after treatment, a paired-sample *t*-test was employed. Differences were considered statistically significant at *p* < 0.05 (indicated by *) and highly significant at *p* < 0.01 (indicated by **).

## 5. Conclusions

In summary, this study demonstrates that *R. palustris* gh32, *B. cereus* HL7, and *B. safensis* X64 achieve efficient degradation and detoxification of indigoid and azo dyes through a highly coordinated, spatiotemporally ordered redox enzyme system. The degradation of azo dyes initiates with the specific reductive cleavage of the azo bond by AZR, followed by an oxidative relay mediated by enzymes such as Lip, Lac, VAO, and Tyr, which collectively drive the hydroxylation and ring-opening reaction. UPLC-Orbitrap-HRMS analysis successfully identified multiple intermediate metabolites generated during the degradation of the four dyes, based on which detailed degradation pathways were proposed, revealing the complete process from chromophore cleavage to the progressive transformation of aromatic rings. Through multi-level biological toxicity assays encompassing microorganisms (*E*. *coli* and *B*. *subtilis*), plant seeds (tobacco and mung bean), and mammalian erythrocytes, it was confirmed that the toxicity of degradation products was significantly reduced, with some treatment groups showing no significant difference from the control group, thereby validating the actual detoxification efficacy of the strains. Enzyme activity assays and RT-qPCR analysis revealed that key oxidoreductases (AZR, Lac, Lip, Tyr, VAO, etc.) were significantly induced under dye stress, and their gene expression levels were highly correlated with changes in enzyme activity, confirming the core driving role of these enzymes in the degradation process. Molecular docking analysis elucidates the structural basis for the specific recognition between enzymes and dyes through hydrogen bonding, salt bridge formation, and hydrophobic interactions and, in conjunction with binding affinity differences, provides molecular-level evidence for explaining strain-specific substrate preferences and differential degradation efficiencies. These findings lay a solid theoretical and experimental foundation for developing targeted and environmentally friendly microbial technologies for dye wastewater treatment.

## Figures and Tables

**Table 1:** The decolorization effects of each strain on the dyes and the reaction parameters.

Dye	Dye Conc. (mg/L)	Strain	Reactive Parameters			Oxygen Supply	Addnl. Subst.	Decolorization Ratio (%) *
			Time (h)	Temp. (°C)	pH			
IC	50	*R. palustris* gh32	6	30	7	aerobic	-	72.21 ± 5.84
RB5	150	*R. palustris* gh32	72	37	7	microaerobic	-	82.54 ± 2.34
DBG	50	*B. cereus* HL7	96	37	7	hypoxia	-	55.16 ± 2.96
DB15	100	*B. safensis* X64	96	37	7	hypoxia	-	67.48 ± 7.15
DB15	100	*B. safensis* X64	96	37	7	hypoxia	sodium citrate	83.17 ± 0.10
DB15	100	*B. safensis* X64	96	37	7	hypoxia	yeast extract	78.13 ± 0.12
IC	50	*B. cereus* HL7	120	37	7	aerobic	-	32.14 ± 3.85
IC	50	*B. cereus* HL7	120	37	7	aerobic	FeCl_3_	87.06 ± 0.47

Note: * Values are a mean of three experiments ± standard deviation. RB5: reactive black 5; DBG: direct black G; DB15: direct blue 15; Conc.: concentration; Temp.: temperature; Addnl. Subst.: additional substances.

**Table 2:** Analysis of protein primary structure.

Uniport ID	Number of Amino Acids	Molecular Weight (kDa)	Theoretical pI	Instability Index	Aliphatic Index	Grand Average of Hydropathicity
Q812W6	272	30.12	5.83	39.44	84.93	−0.254
A5A677	287	31.8	5.56	34.57	76.41	−0.269
B7JU04	247	28.5	5.47	32.58	68.3	−0.651
Q2IY37	416	45.02	6.35	34.93	77.91	−0.25
Q73CJ5	213	24.45	4.89	37.01	89.07	−0.415
O32224	211	23.27	5.26	34.81	69.43	−0.337
Q215Z0	202	21.04	6.29	27.3	106.88	0.372

**Table 3:** Analysis of protein secondary structure.

Uniport ID	α-Helix (%)	β-Sheet (%)	Random Coil (%)
Q812W6	22.79	26.47	50.74
A5A677	23.08	24.48	52.45
B7JU04	33.2	9.31	57.49
Q2IY37	19.95	15.14	64.9
Q73CJ5	48.83	14.08	37.09
O32224	46.92	14.69	38.39
Q215Z0	47.03	14.85	38.12

**Table 4:** Analysis of protein tertiary structure.

Uniport ID	ERRAT	Verify 3D (%)	PROCHECK (%)			
			Favored ^1^	Allowed ^2^	General ^3^	Disallowed ^4^
Q812W6	87.149	76.78	91	8.1	0.4	0.4
A5A677	95.131	80.73	92.5	7.1	0.4	0
B7JU04	94.561	91.5	91.5	7.1	1.4	0
Q2IY37	94.679	94.71	89.5	9.9	0.3	0.3
Q73CJ5	93.069	84.04	89.3	9.6	0.5	0.5
O32224	97.5	97.63	91.7	8.3	0	0
Q215Z0	98.404	59.41	91.8	8.2	0	0

^1^ Favored: residues in most favored regions. ^2^ Allowed: residues in additional allowed regions. ^3^ General: residues in generously allowed regions. ^4^ Disallowed: residues in disallowed regions.

**Table 5:** Summary of results using AutoDock Vina docking enzyme and small molecules.

Enzyme-Ligand Complex	Binding Energy (kcal/mol)	Hydrogen Bond Residues Involved	Hydrogen Bond Distance (Å)	Hydrophobic Interaction	Salt Bridge
HL7-Lac					
(Q812W61) ^1^					
IC	−7.8	**His47**	3.07	**Ala123, Asp124**	**His47**
		**Gln78**	3.03	**Cys125, Trp145**	**His80**
		**His80**	2.96	**His250, Arg259**	**His142**
		**Tyr99**	(3.10/3.12)		
		**Cys121**	3.31		
		**Tyr183**	2.91		
		**Arg251**	3.35		
DBG	−7.2	**Tyr99**	2.83	**His47, Gln78**	Arg188
		Arg188	2.97	**His80, Cys121**	**His250**
		**His250**	3.1	**Ala123, Asp124**	
		**Arg259**	3.09	**Cys125, His142**	
				**Trp145**, Val184	
				Asp186, **Arg251**	
				Gln254	
X64-Lac					
(A5A677) ^2^					
DB15	−8.7	**His63**	3.12	**Leu62,** Ala68	**His96**
		**His96**	2.79	**Asp93, Gln94**	**His158**
		**Tyr115**	3.26	Tyr95, His114	**His268**
		**Ala139**	3.17	Asp116, **Phe138**	**Arg277**
		**His158**	2.89	**Tyr200, Arg277**	
		**His268**	3.21		
HL7-Tyr					
(B7JU04) ^1^					
IC	−6.7	**Gln48**	2.93	**Phe42, Phe43**	-
		**Asn149**	(2.91/3.00)	**His47, Gln135**	
				**His152, Asn153**	
				**Ala160**	
DBG	−7.5	Asn110	(3.13/3.15)	**Phe42, His47**	-
		Gly139	2.98	**Gln48**, Phe112	
		**Glu146**	(3.03/3.05/3.03/3.04)	**Thr137**, Ser138	
			3.11	Ala143, **His148**	
		**Asn153**		**Asn149**, Gly150	
				**His152**	
gh32-Tyr					
(Q2IY37) ^3^					
IC	−7.2	**Arg57**	(2.93/3.09/3.21)	**Val23**, Gln34	**Arg57**
		**Thr371**	3	**Phe79, Thr372**	
		Leu390	3.06	Ala392, **Asp394**	
		**Pro393**	2.89	**Pro395**	
RB5	−7.6	**Glu10**	2.94	Asp6, **Ile7**	**Arg57**
		**Thr21**	3.33	**Pro8, Gly9**	
		**Gln24**	(2.74/2.87/3.16)	**Gly19, Val23**	
		**Gly397**	3.33	**Arg57, Phe79**	
		**Phe398**	3.29	**Thr371, Pro395**	
				**Ala399**	
HL7-AZR					
(Q73CJ5) ^1^					
DBG	−7.7	**His10**	2.9	Pro11, **Lys12**	-
		Tyr47	3.14	**Ser18, Pro100**	
		**His102**	3.14	**Leu101**, Ile106	
		**Asn103**	3.11	Pro107, **Ala146,**	
		**Gly148**	2.98	**Ser147, Tyr151**	
				**Thr187**	
X64-AZR					
(O32224) ^2^					
DB15	−7.9	**His10**	3.03	**Thr16, Ser17**	**His10**
		**Tyr18**	3.04	**Pro101, Leu102**	
		**Trp103**	3.24	**Asn104, Phe105**	
		**Tyr151**	2.96	**Ala146, Gly148**	
		**His186**	3.12	**Gly149**, Tyr150	
				**Asn187**, Ala188	
gh32-AZR					
(Q215Z0) ^3^					
RB5	−7.1	His49	2.9	Ile10, **Leu11**	His49
		**Asn95**	3.09	**Pro92, Met93**	**Lys179**
		Thr100	2.71	**Tyr94**, Thr97	
		**Ser137**	3.05	Ile98, Lys103	
		**Gly139**	3.25	Gln101, **Ile178**	
		**Lys179**	2.95	**Arg138**	

^1^ Laccase (Q812W6), tyrosinase (B7JU04), azoreductase (Q73CJ5) from *B. cereus* HL7. ^2^ Laccase (A5A677), and azoreductase (O32224) from *B. safensis* X64. ^3^ Tyrosinase (Q2IY37), and azoreductase (Q215Z0) from *R. palustris* gh32. Residues located within the active site are indicated in boldface. For each docking result, they are listed starting with the one with the lowest serial number.

## Data Availability

The data presented in this study are available in the article.

## Supplementary Materials

The following supporting information can be downloaded at: https://www.mdpi.com/article/10.3390/ijms27072980/s1.
