# Supplementary material for: Elucidating the Multi-Enzymatic Mechanism of Bacterial Decolorization of Azo and Indigoid Dyes: An Integrated Study of Degradation Pathways and Molecular Docking
Source: Int J Mol Sci. 2026 Mar 25;27(7):2980. doi: 10.3390/ijms27072980 (PMC13073463; doi:10.3390/ijms27072980)
Supplement: Supplementary file 1 [file ijms-27-02980-s001.zip › ijms-4160034-supplementary.pdf]

Elucidating the Multi-Enzymatic Mechanism of Bacterial Decolorization of Azo and Indigoid Dyes: An Integrated Study of Degradation Pathways and Molecular Docking

Chunlei Wang [a,b]* , Tongshuai Liu[a], He Song[a], Yang Zhao[a],Haowei Wang[a], Jinshuo Li[a], Jieru Zhang[a], Sijia Wang[a], Yongdi Wang[a], Jixia Wang[a], Shumin Jiang[a], Chengwei Liu [a,b] *

[a] Key Laboratory of National Forestry and Grassland Administration on Chinese Herbal Medicine, College of Life Science, Northeast Forestry University, Harbin 150040, China;

[b] State Key Laboratory of Utilization of Woody Oil Resource, Northeast Forestry University, Harbin 150040, China.

Table S1. UPLC- Orbitrap-HRMS data for indigo carmine and the products of its degradation

Serial Number	Chemical Formula	Compound Name	Mol wt.	Adduct of the Substance	Experimental m/z of the Adduct	Theoretical m/z of the Adduct	Difference of m/z (ppm)	Retention Time (min)	CAS/CID
1	C16H8N2Na2O8S2	indigo carmine	465.95175	[M-2Na]2-	209.98621	209.98665	2.11	5.42	860-22-0
2	C16H10N2O2	(2E)-2-(3-oxo-1H-indol-2-ylidene)-1H-indol-3-one	262.07423	[M+H]+	263.08142	263.08150	0.30	8.66	/5318432
3	C9H7NO	2-methylidene-1H-indol-3-one	145.05276	[M+H]+	146.06000	146.06004	0.27	7.09	/20614798
4	C8H5NO2	isatin	147.03203	[M+H]+	148.03915	148.03931	1.08	21.2	91-56-5
5	C7H7NO	2-aminobenzaldehyde	121.05276	[M+H]+	122.0602	122.06004	1.31	21.04	529-23-7
6	C7H5NO3	2-nitrobenzaldehyde	151.02694	[M+H]+	152.03407	152.03422	0.99	21.6	552-89-6
7	C7H5NO4	2-nitrobenzoic acid	167.02186	[M+H]+	168.02892	168.02913	1.25	3.79	552-16-9
8	C6H5NO2	nitrobenzene	123.03203	[M+H]+	124.0394	124.03931	0.73	1.6	98-95-3
9	C6H5NO3	3-nitrophenol	139.02694	[M+H]+	140.03435	140.03422	0.93	1.55	554-84-7
10	C7H7NO2	2-aminobenzoic acid	137.04768	[M+H]+	138.05490	138.05496	0.43	4.7	118-92-3
11	C6H7NO2	4-aminobenzene-1,3-diol	125.04768	[M+H]+	126.05494	126.05496	0.16	1.6	13066-95-0
12	C7H7NO5	2-amino-3,4,5-trihydroxybenzoic acid	185.03242	[M+H]+	186.03925	186.03970	2.42	3.08	/56978624
13	C7H15NO	(1-aminocyclohexyl)methanol	129.11536	[M+H]+	130.12286	130.12264	1.69	11.73	4313-56-8
14	C8H9NO2	2-aminophenylacetic acid	151.06333	[M+H]+	152.07054	152.07061	0.46	5.88	3342-78-7
15	C8H5NO5S	2,3-dioxo-5-indolinesulfonic acid	226.98884	[M-H]-	225.98122	225.98157	1.56	5.32	7313-70-4
16	C8H7NO6S	2-(2-amino-5-sulfophenyl)-2-oxoacetic acid	244.99941	[M-H]-	243.99194	243.99213	0.78	1.58	/15250315
17	C7H7NO5S	2-amino-5-sulfobenzoic acid	217.00449	[M-H]-	215.9968	215.99722	1.95	1.58	3577-63-7
18	C8H9NO3S	2,3-dihydro-1H-indole-5-sulfonic acid	199.03031	[M+H]+	200.03845	200.03759	4.30	7.8	98487-89-9

Table S2. UPLC- Orbitrap-HRMS data for RB5 and the products of its degradation

Serial Number	Chemical Formula	Compound Name	Mol wt.	Adduct of the Substance	Experimental m/z of the Adduct	Theoretical m/z of the Adduct	Difference of m/z (ppm)	Retention Time (min)	CAS /CID
1	C26H25N5O19S6	4-imino-5-oxo-3,6-bis[[4-(2-sulfooxyethylsulfonyl) phenyl] hydrazinylidene]naphthalene-2,7-disulfonic acid	902.94680	[M-3H]3-	299.9736	299.97499	4.65	3.8	/28266
2	C8H11NO6S2	2-[(4-aminophenyl) sulfonyl]ethyl hydrogen sulfate	281.00278	[M-H]-	279.99573	279.99550	0.82	4.09	2494-89-5
3	C18H18N4O13S4	3,5-diamino-4-hydroxy-6-[[4-(2-sulfooxyethylsulfonyl) phenyl]diazenyl] naphthalene-2,7-disulfonic acid	625.97532	[M-H]-	624.96863	624.96804	0.95	8.13	/136612193
4	C8H11NO3S	2-((p-aminophenyl)sulphonyl)ethanol	201.04596	[M-H]-	200.03836	200.03869	1.65	6.06	5246-58-2
5	C8H9NO2S	4-ethenylsulfonylaniline	183.03540	[M+H]+	184.04266	184.04268	0.11	6.25	25781-90-2
6	C6H7NO3S	4-aminobenzenesulfonic acid	173.01466	[M-H]-	172.00656	172.00739	4.83	1.68	121-57-3
7	C7H8O2S	methyl phenyl sulfone	156.02450	[M+H]+	157.03253	157.03178	4.78	4.47	3112-85-4
8	C4H4O3	4-oxobut-2-enoic acid	100.01604	[M-H]-	99.00886	99.00877	0.91	4.89	1575-59-3
9	C10H11N3O7S2	3,4,6-triamino-5-hydroxynaphthalene-2,7-disulfonic acid	349.00384	[M-H]-	347.99820	347.99656	4.73	10.24	69762-07-8
10	C10H8O5S	6,7-dihydroxynaphthalene-2-sulfonic acid	240.00924	[M-H]-	239.00200	239.00197	0.13	11.16	92-27-3
11	C10H8O2	naphthalene-1,2-diol	160.05243	[M-H]-	159.04442	159.04515	4.59	7.27	574-00-5
12	C10H6O2	naphthalene-1,2-dione	158.03678	[M+H]+	159.04395	159.04406	0.69	7.00	524-42-5
13	C11H9NO2	8-amino-7-methylnaphthalene-1,2-dione	187.06333	[M+H]+	188.07063	188.07061	0.11	5.23	/12324882
14	C8H4O3	2-benzofuran-1,3-dione	148.01604	[M+H]+	149.02327	149.02332	0.34	12.21	85-44-9
15	C9H8O3	2-acetylbenzoic acid	164.04734	[M-H]-	163.03926	163.04007	4.97	9.63	577-56-0
16	C10H7NNa2O7S2	disodium;4-amino-5-hydroxynaphthalene-2,7-disulfonate	362.94593	[M-2Na]2-	158.48297	158.48374	4.87	1.89	3963-80-2
17	C10H9NO3S	5-amino-2-naphthalenesulfonic acid	223.03031	[M-H]-	222.02292	222.02304	0.54	2.53	119-79-9
18	C10H9NO	1-amino-2-naphthol	159.06841	[M-H]-	158.06036	158.06114	4.94	8.78	2834-92-6
19	C10H9NO2	8-aminonaphthalene-1,2-diol	175.06333	[M+H]+	176.07051	176.07061	0.57	9.26	/129857117
20	C10H9NO3	8-aminonaphthalene-1,2,7-triol	191.05824	[M-H]-	190.05040	190.05097	2.98	7.68	/129938001
21	C10H10N2O	2,8-diaminonaphthalen-1-ol	174.07931	[M+H]+	175.08664	175.08659	0.29	7.24	/82254060
22	C10H7NO2	8-aminonaphthalene-1,2-dione	173.04768	[M+H]+	174.05495	174.05496	0.06	10.52	/91533485
23	C10H11NO4	2-amino-6-(2-carboxyethyl)benzoic acid	209.06881	[M-H]-	208.06133	208.06153	0.96	5.15	/119017811
24	C10H9NO4	2-amino-4-(2-carboxyethenyl)benzoic acid	207.05316	[M+H]+	208.06052	208.06043	0.43	4.82	/6612013
25	C16H13N3O8S2	4-amino-5-hydroxy-3-[(4-hydroxyphenyl) diazenyl]naphthalene-2,7-disulfonic acid	439.01441	[M-H]-	438.00815	438.00713	2.33	9.58	/136676244

Table S3. Comparison of RB5 degradation products among different treatment groups mediated by strain gh32

Serial Number	Chemical Formula	Compound Name	DP-24 h	DP-5 d
1	$C_{26}H_{25}N_5O_{19}S_6$	4-imino-5-oxo-3,6-bis[[4-(2-sulfooxyethylsulfonyl) phenyl] hydrazinylidene]naphthalene-2,7-disulfonic acid	+	+
2	$C_8H_{11}NO_6S_2$	2-[(4-aminophenyl) sulfonyl]ethyl hydrogen sulfate	+	-
3	$C_{18}H_{18}N_4O_{13}S_4$	3,5-diamino-4-hydroxy-6-[[4-(2-sulfooxyethylsulfonyl) phenyl]diazenyl] naphthalene-2,7-disulfonic acid	+	+
4	$C_8H_{11}NO_3S$	2-((p-aminophenyl)sulphonyl)ethanol	+	+
5	$C_8H_9NO_2S$	4-ethenylsulfonylaniline	+	-
6	$C_6H_7NO_3S$	4-aminobenzenesulfonic acid	+	+
7	$C_7H_8O_2S$	methyl phenyl sulfone	+	+
8	$C_4H_4O_3$	4-oxobut-2-enoic acid	-	+
9	$C_{10}H_{11}N_3O_7S_2$	3,4,6-triamino-5-hydroxynaphthalene-2,7-disulfonic acid	+	-
10	$C_{10}H_8O_5S$	6,7-dihydroxynaphthalene-2-sulfonic acid	-	+
11	$C_{10}H_8O_2$	naphthalene-1,2-diol	+	+
12	$C_{10}H_6O_2$	naphthalene-1,2-dione	+	+
13	$C_{11}H_9NO_2$	8-amino-7-methylnaphthalene-1,2-dione	+	+
14	$C_8H_4O_3$	2-benzofuran-1,3-dione	+	+
15	$C_9H_8O_3$	2-acetylbenzoic acid	+	+
16	$C_{10}H_7NNa_2O_7S_2$	disodium;4-amino-5-hydroxynaphthalene-2,7-disulfonate	+	-
17	$C_{10}H_9NO_3S$	5-amino-2-naphthalenesulfonic acid	+	-
18	$C_{10}H_9NO$	1-amino-2-naphthol	+	+
19	$C_{10}H_9NO_2$	8-aminonaphthalene-1,2-diol	+	+
20	$C_{10}H_9NO_3$	8-aminonaphthalene-1,2,7-triol	+	+
21	$C_{10}H_{10}N_2O$	2,8-diaminonaphthalen-1-ol	+	+
22	$C_{10}H_7NO_2$	8-aminonaphthalene-1,2-dione	+	+
23	$C_{10}H_{11}NO_4$	2-amino-6-(2-carboxyethyl)benzoic acid	+	+
24	$C_{10}H_9NO_4$	2-amino-4-(2-carboxyethenyl)benzoic acid	+	+
25	$C_{16}H_{13}N_3O_8S_2$	4-amino-5-hydroxy-3-[(4-hydroxyphenyl) diazenyl]naphthalene-2,7-disulfonic acid	+	+

Note: DP: degradation product; DP-24 h/5d: DP after 24 h /5d of static treatment.

Table S4. UPLC- Orbitrap-HRMS data for DBG and the products of its degradation

Serial Number	Chemical Formula	Compound Name	Mol wt.	Adduct of the Substance	Experimental m/z of the Adduct	Theoretical m/z of the Adduct	Difference of m/z (ppm)	Retention Time (min)	CAS /CID
1	C34H29N13O7S2	3,6-bis[[4-[(2,4-diaminophenyl)diazenyl] phenyl]hydrazinylidene]-4-imino-5-oxonaphthalene-2,7-disulfonic acid	795.17543	[M+2H]2+	398.59326	398.59499	4.35	7.29	/22941
2	C6H8N2	p-phenylenediamine	108.06875	[M+H]+	109.07558	109.07602	4.04	1.23	106-50-3
3	C10H10N2	1,7-naphthalenediamine	158.08440	[M+H]+	159.09120	159.09167	2.96	2.27	2243-64-3
4	C10H11N3O7S2	3,4,6-triamino-5-hydroxynaphthalene-2,7-disulfonic acid	349.00384	[M-H]-	347.99557	347.99656	2.85	13.52	69762-07-8
5	C6H7N	aniline	93.05785	[M+H]+	94.06490	94.06513	2.45	1.69	62-53-3
6	C10H11N3O	2,7,8-triaminonaphthalen-1-ol	189.09021	[M+H]+	190.09659	190.09749	4.74	2.97	/129853186
7	C10H9N	2-naphthylamine	143.07350	[M+H]+	144.08011	144.08078	4.65	5.45	91-59-8
8	C10H9NO	1-amino-2-naphthol	159.06841	[M+H]+	160.07489	160.07569	5.00	1.31	2834-92-6
9	C10H8O2	naphthalene-1,2-diol	160.05243	[M-H]-	159.04446	159.04515	4.34	5.59	574-00-5
10	C10H8O3	naphthalene-1,2,4-triol	176.04734	[M-H]-	175.03984	175.04007	1.31	0.88	13302-67-5
11	C10H9NO3	8-aminonaphthalene-1,2,7-triol	191.05824	[M+H]+	192.06462	192.06552	4.69	1.31	/129938001
12	C10H9NO2	8-aminonaphthalene-1,2-diol	175.06333	[M+H]+	176.06981	176.07061	4.55	1.49	/129857117
13	C10H7NO2	8-aminonaphthalene-1,2-dione	173.04768	[M+H]+	174.05464	174.05496	1.84	5.92	/91533485
14	C11H9NO2	8-amino-7-methylnaphthalene-1,2-dione	187.06333	[M+H]+	188.06998	188.07061	3.35	3.7	/12324882
15	C10H9NO4	2-amino-4-(2-carboxyethenyl)benzoic acid	207.05316	[M+H]+	208.05948	208.06043	4.57	2.79	/6612013
16	C10H9NO7S2	4-amino-5-hydroxy-2,7-naphthalenedisulfonic acid	318.98204	[M+H]+	319.98813	319.98932	3.73	6.85	90-20-0
17	C10H9NO3S	5-amino-2-naphthalenesulfonic acid	223.03031	[M+H]+	224.03856	224.03759	4.33	2.18	119-79-9
18	C7H7NO2	4-aminobenzoic acid	137.04768	[M+H]+	138.05437	138.05496	4.28	5.55	150-13-0
19	C10H8O5S	6,7-dihydroxynaphthalene-2-sulfonic acid	240.00924	[M-H]-	239.00188	239.00197	0.38	1.41	92-27-3
20	C8H6O4	phthalic acid	166.02661	[M-H]-	165.01862	165.01933	4.30	4.98	88-99-3
21	C5H8O4	4-hydroxy-2-oxopentanoic acid	132.04226	[M+H]+	133.04909	133.04954	3.38	2.36	3318-73-8

Table S5. Comparison of DBG degradation products among different treatment groups mediated by strain HL7

Serial Number	Chemical Formula	Compound Name	DP-7 d	DP- M -5 d	DP-AZR-2 d
1	$C_{34}H_{29}N_{13}O_7S_2$	3,6-bis[[4-[(2,4-diaminophenyl)diazenyl] phenyl]hydrazinylidene]-4-imino-5-oxonaphthalene-2,7-disulfonic acid	+	+	+
2	$C_6H_8N_2$	p-phenylenediamine	+	+	+
3	$C_{10}H_{10}N_2$	1,7-naphthalenediamine	-	-	+
4	$C_{10}H_{11}N_3O_7S_2$	3,4,6-triamino-5-hydroxynaphthalene-2,7-disulfonic acid	-	+	-
5	C_6H_7N	aniline	+	+	+
6	$C_{10}H_{11}N_3O$	2,7,8-triaminonaphthalen-1-ol	+	+	-
7	$C_{10}H_9N$	2-naphthylamine	-	+	+
8	$C_{10}H_9NO$	1-amino-2-naphthol	-	+	+
9	$C_{10}H_8O_2$	naphthalene-1,2-diol	-	-	+
10	$C_{10}H_8O_3$	naphthalene-1,2,4-triol	-	-	+
11	$C_{10}H_9NO_3$	8-aminonaphthalene-1,2,7-triol	+	-	+
12	$C_{10}H_9NO_2$	8-aminonaphthalene-1,2-diol	+	+	+
13	$C_{10}H_7NO_2$	8-aminonaphthalene-1,2-dione	-	-	+
14	$C_{11}H_9NO_2$	8-amino-7-methylnaphthalene-1,2-dione	+	+	+
15	$C_{10}H_9NO_4$	2-amino-4-(2-carboxyethenyl)benzoic acid	-	-	+
16	$C_{10}H_9NO_7S_2$	4-amino-5-hydroxy-2,7-naphthalenedisulfonic acid	+	-	-
17	$C_{10}H_9NO_3S$	5-amino-2-naphthalenesulfonic acid	+	+	-
18	$C_7H_7NO_2$	4-aminobenzoic acid	-	-	+
19	$C_{10}H_8O_5S$	6,7-dihydroxynaphthalene-2-sulfonic acid	-	+	+
20	$C_8H_6O_4$	phthalic acid	+	+	+
21	$C_5H_8O_4$	4-hydroxy-2-oxopentanoic acid	+	-	+

Note: DP:degradation product; DP-7 d: DP after 7 d by static treatment with sealing film; DP-M-5 d: DP after 5 d by static treatment with sealing film added sodium 2-anthraquinonesulfonate as redox mediator.

Table S6. UPLC- Orbitrap-HRMS data for DB15 and the products of its degradation

Serial Number	Chemical Formula	Compound Name	Mol wt.	Adduct of the Substance	Experimental m/z of the Adduct	Theoretical m/z of the Adduct	Difference of m/z (ppm)	Retention Time (min)	CAS /CID
1	C34H24N6Na4O16S4	Direct blue 15	991.97224	[M-Na]-	968.97913	968.98302	4.02	8.09	2429-74-5
2	C10H9NO7S2	4-amino-5-hydroxy-2,7-naphthalenedisulfonic acid	318.98204	[M+H]+	319.98788	319.98932	4.51	6.72	90-20-0
3	C14H16N2O2	3,3'-dimethoxybenzidine	244.12118	[M+H]+	245.12746	245.12845	4.04	5.27	119-90-4
4	C10H8O7S2	1-naphthol-3,6-disulfonic acid	303.97114	[M-H]-	302.96527	302.96387	4.64	9.39	578-85-8
5	C10H8O	1-naphthol	144.05751	[M-H]-	143.04982	143.05024	2.94	2.46	90-15-3
6	C10H6O2	1,2-naphthoquinone	158.03678	[M+H]+	159.04332	159.04406	4.65	2.5	524-42-5
7	C10H9NO	8-amino-1-naphthol	159.06841	[M+H]+	160.07494	160.07569	4.69	10.63	2834-91-5
8	C12H12N2	benzidine	184.10005	[M+H]+	185.10667	185.10733	3.57	5.72	92-87-5
9	C8H11NO	2-methoxy-4-methylaniline	137.08406	[M+H]+	138.09071	138.09134	4.57	2.62	39538-68-6
10	C7H9NO	O-anisidine	123.06841	[M+H]+	124.07511	124.07569	4.68	2.33	90-04-0
11	C7H8O	anisole	108.05751	[M-H]-	107.05011	107.05024	1.21	6.5	100-66-3
12	C6H7N	aniline	93.05785	[M+H]+	94.06472	94.06513	4.36	1.61	62-53-3
13	C6H7NO	2-aminophenol	109.05276	[M+H]+	110.0595	110.06004	4.91	1.35	95-55-6
14	C7H8N2O	(3-methoxyphenyl)diazole	136.06366	[M+H]+	137.07031	137.07094	4.60	14.54	/67050871
15	C14H16N2	3,3'-dimethylbenzidine	212.13135	[M+2H]2+	107.07259	107.07295	3.36	4.2	119-93-7
16	C14H14O2	3,3'-dimethoxybiphenyl	214.09938	[M+H]+	215.10562	215.10666	4.84	7.34	6161-50-8
17	C6H6O2	catechol	110.03678	[M-H]-	109.02936	109.02950	1.28	9.71	120-80-9
18	C6H4O2	1,2-benzoquinone	108.02113	[M+H]+	109.02788	109.02841	4.86	0.28	583-63-1
19	C4H4O4	But-2-enedioic acid	116.01096	[M-H]-	115.00378	115.00368	0.87	4.48	6915-18-0

Table S7. Comparison of DB15 degradation products among different treatment groups mediated by strain X64

Serial Number	Chemical Formula	Compound Name	DP-7 d	DP-5+2 d	DP-YE-7 d	DP-SC-7 d
1	C34H24N6Na4O16S4	Direct blue 15	+	+	+	+
2	C10H9NO7S2	4-amino-5-hydroxy-2,7-naphthalenedisulfonic acid	-	-	+	-
3	C14H16N2O2	3,3'-dimethoxybenzidine	+	+	+	+
4	C10H8O7S2	1-naphthol-3,6-disulfonic acid	-	+	+	+
5	C10H8O	1-naphthol	-	+	-	-
6	C10H6O2	1,2-naphthoquinone	-	+	+	+
7	C10H9NO	8-amino-1-naphthol	-	+	-	+
8	C12H12N2	benzidine	-	+	-	+
9	C8H11NO	2-methoxy-4-methylaniline	-	+	-	+
10	C7H9NO	O-anisidine	-	+	-	+
11	C7H8O	anisole	-	-	-	+
12	C6H7N	aniline	+	-	+	+
13	C6H7NO	2-aminophenol	-	-	+	+
14	C7H8N2O	(3-methoxyphenyl)diazole	-	+	+	-
15	C14H16N2	3,3'-dimethylbenzidine	-	+	-	-
16	C14H14O2	3,3'-dimethoxybiphenyl	-	-	-	+
17	C6H6O2	catechol	-	-	-	+
18	C6H4O2	1,2-benzoquinone	+	+	+	+
19	C4H4O4	But-2-enedioic acid	-	-	+	-

Note: DP: degradation product; DP-7 d: DP after 7 d by static treatment with sealing film; DP-5+2 d: DP after 5 days of static treatment with sealing film followed by 2 days of agitation; DP-SC-7d: DP after 7 d by static treatment with sealing film added sodium citrate; DP-YE-7d: DP after 7 d by static treatment with sealing film added yeast extract.

Table S8. Oxidoreductase specific primers and target fragment sizes

Primer Name	Primer Sequence (5′-3′)	Amplicon length (bp)
reference gene:		
16S rRNA-1 F	TACGGCTACCTTGTTACGACTT	184
16S rRNA-1 R	GAAGCTGGAATCGCTAGTAATC	
For HL7:		
cotA-1 F	GCACATTGTGGTATCACGATACT	155
cotA-2 R	AACAGTGCGCCATCCTCCT	
melA-1 F	GTGGTACAACAGGTGCAGAT	151
melA-2 R	CAGTCTACTGGATCTAACGAATAGT	
azoR-1 F	TGTCTGGTGGTGGGATTTACC	230
azoR-2 R	CCAAGTCAGCAAGAGTTCTG	
ndh-1 F	GAGTACCTGCGGATTGGATTG	169
ndh-2 R	CCACCAGTTGAACCTGTAACG	
For X64:		
azoR-3 F	CATCCTTGGGATGGAAGCT	126
azoR-4 R	GATCAGGCGTTTCTCCTTTAT	

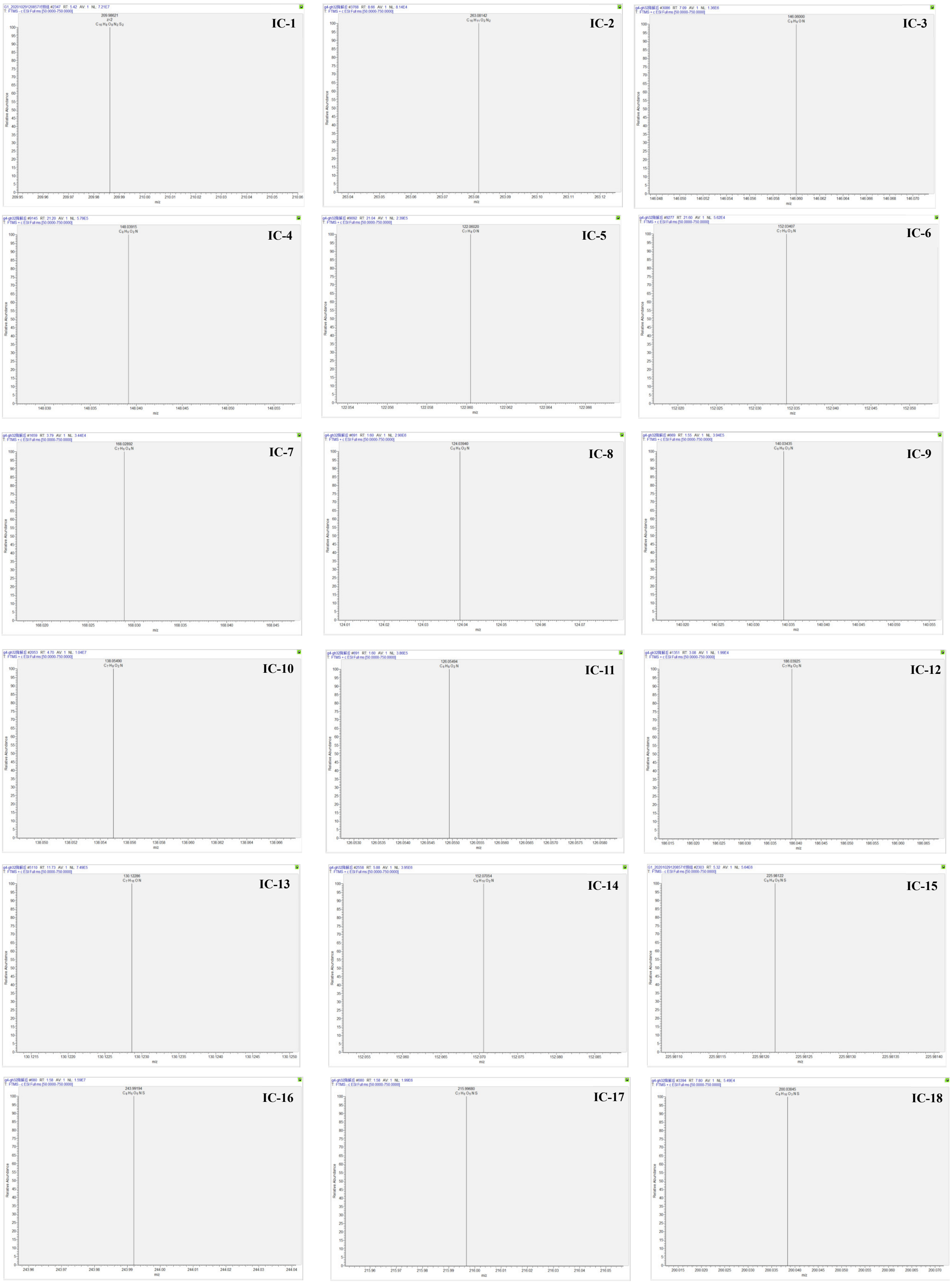

Figure S1. Mass spectra of IC and its biodegradation intermediates by strain gh32.

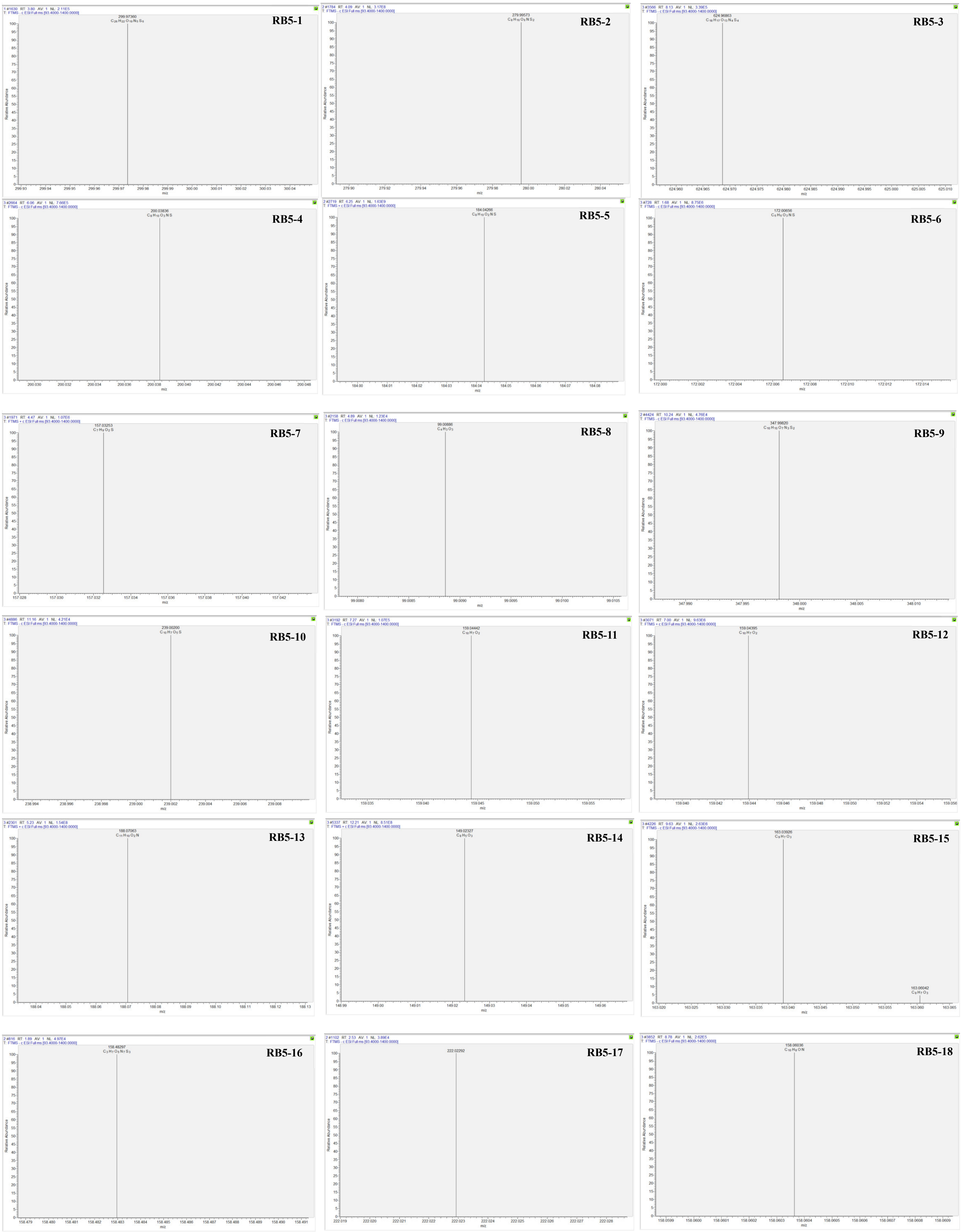

Figure S2. Mass spectra of RB5 and its biodegradation intermediates by strain gh32.

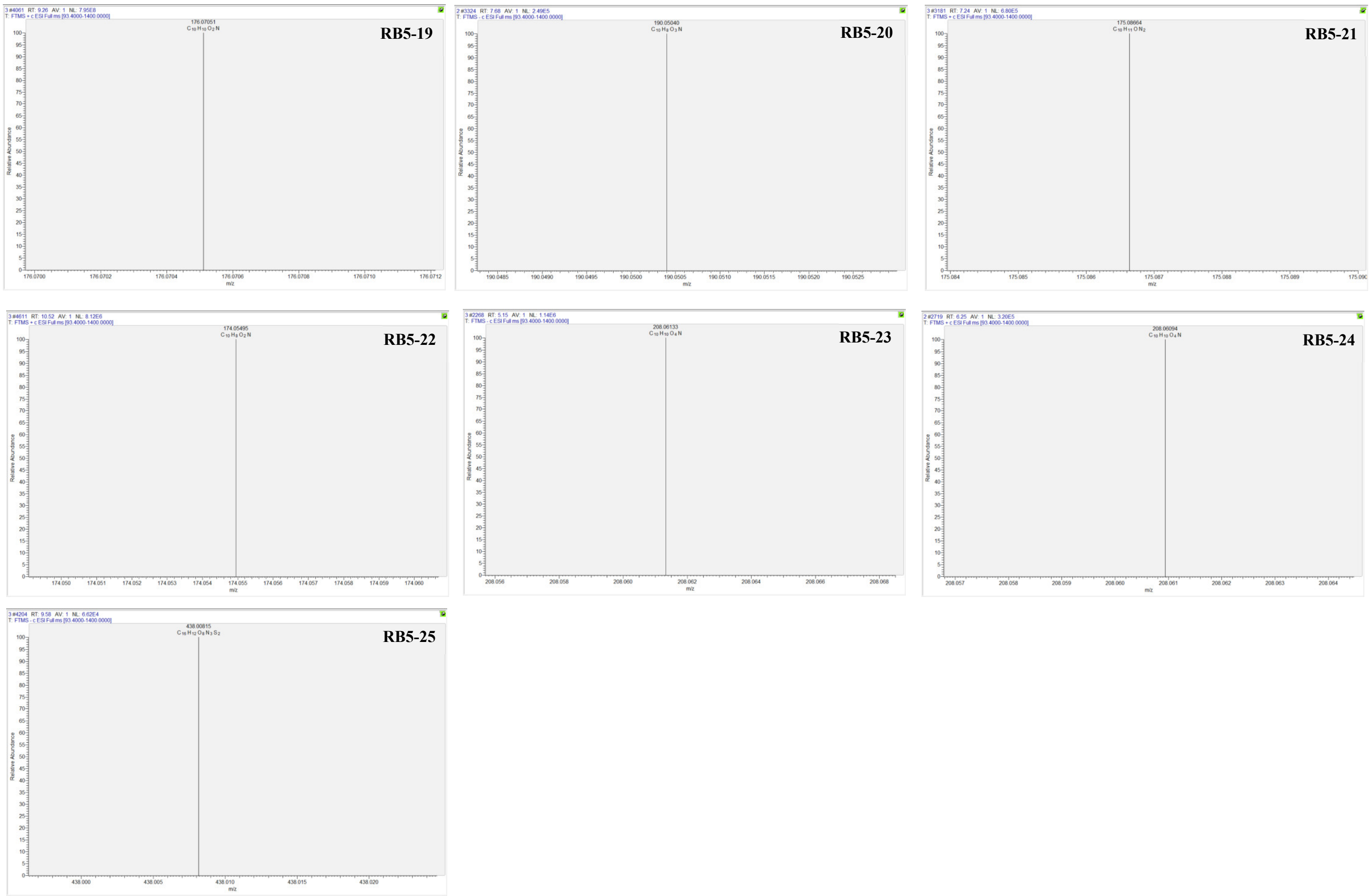

Figure S2. (continued) Mass spectra of RB5 and its biodegradation intermediates by strain gh32.

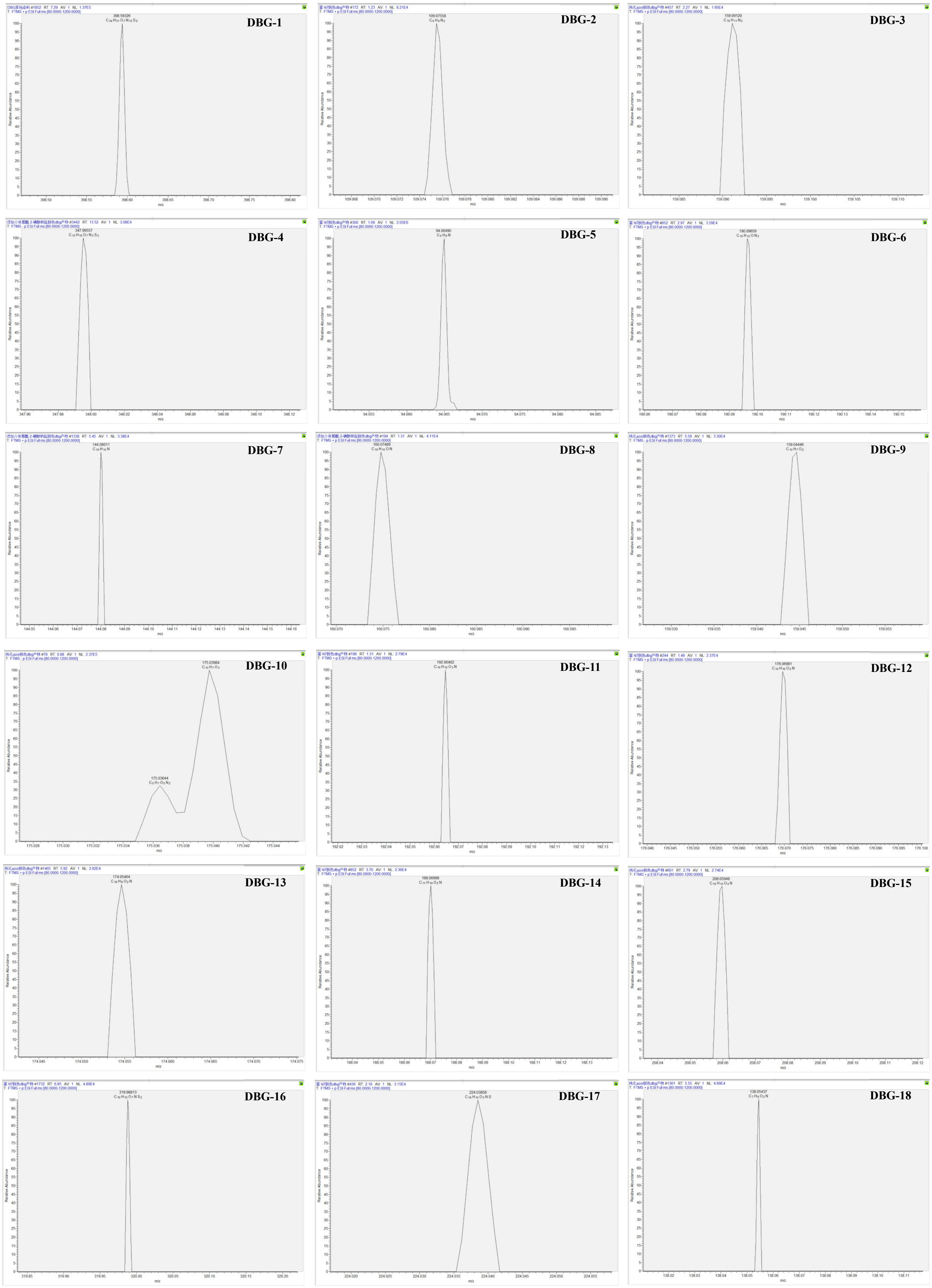

Figure S3. Mass spectra of DBG and its biodegradation intermediates by strain HL7.

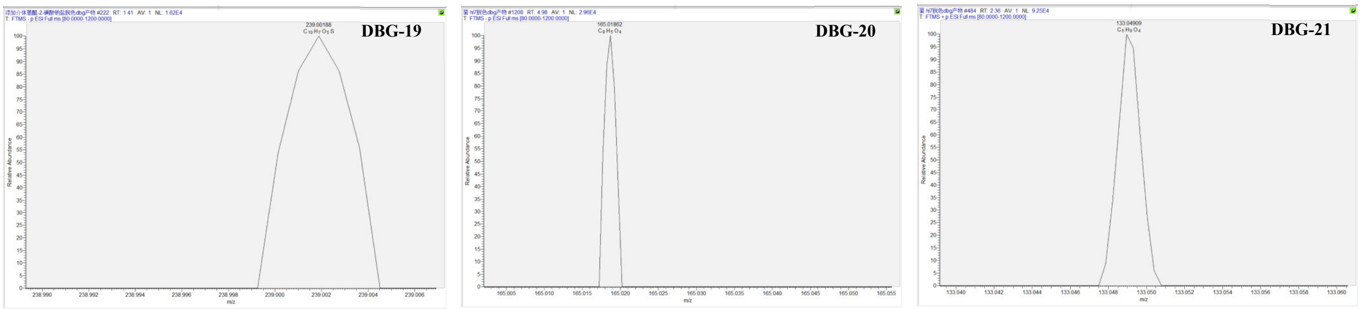

Figure S3. (continued) Mass spectra of DBG and its biodegradation intermediates by strain HL7.

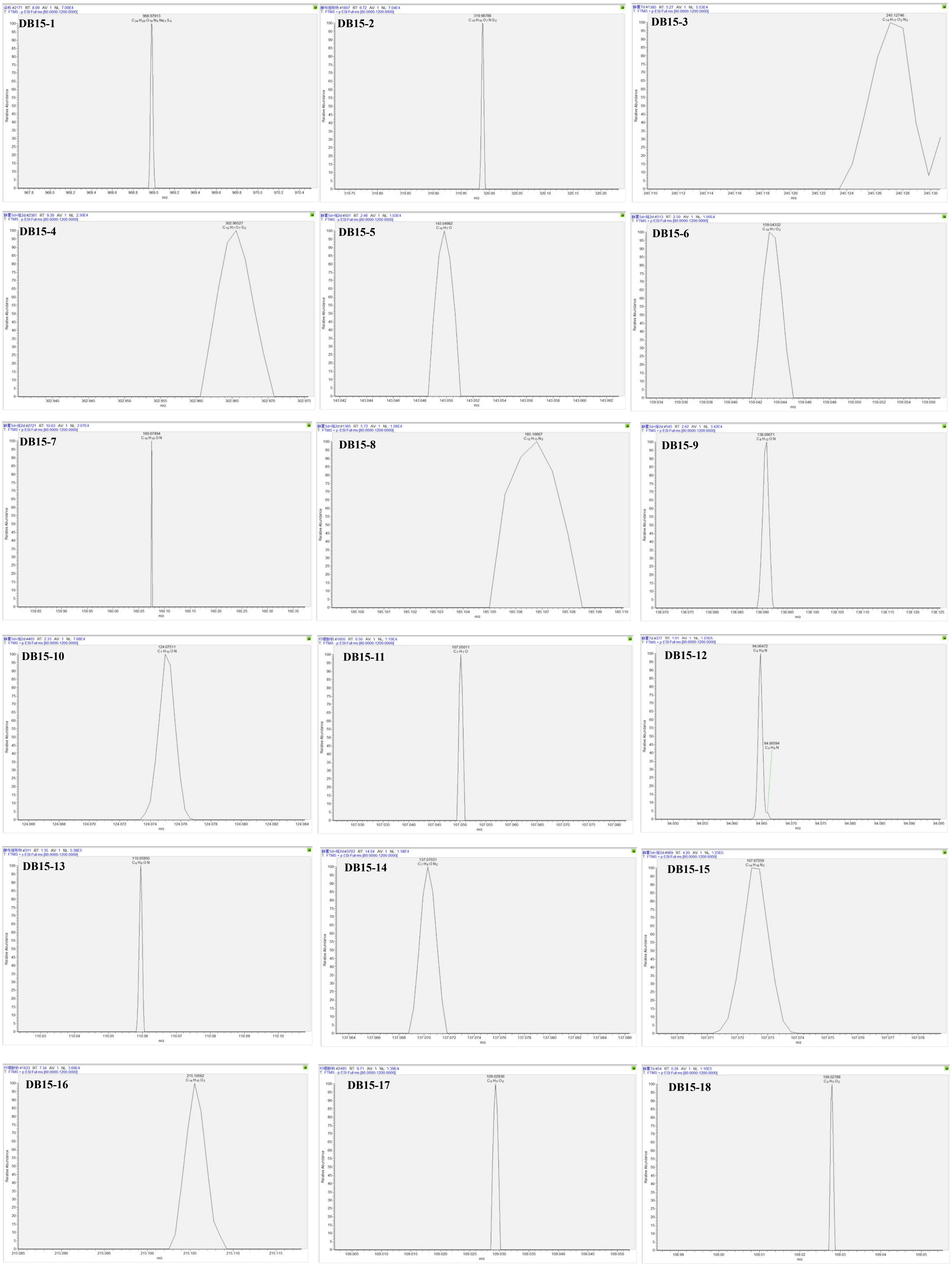

Figure S4. Mass spectra of DB15 and its biodegradation intermediates by strain X64.

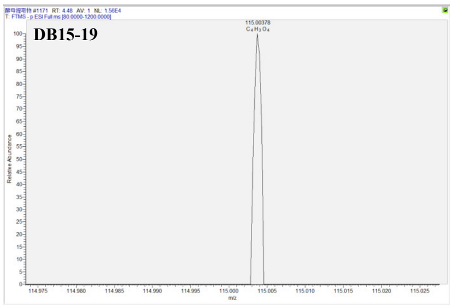

Figure S4. (continued) Mass spectra of DB15 and its biodegradation intermediates by strain X64.

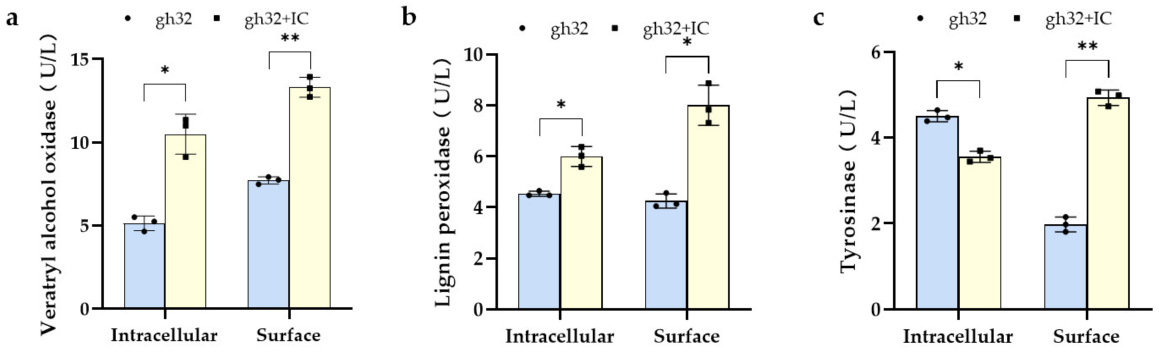

Figure S5. Enzyme activity analysis of strain gh32 in the degradation of indigo carmine (IC). (a) veratryl alcohol oxidase; (b)lignin peroxidase; (c) tyrosinase. The data are analyzed by a paired-sample t-test using means of three experiments. * indicates significant difference at $p < 0.05$. ** indicates significant difference at $p < 0.01$.

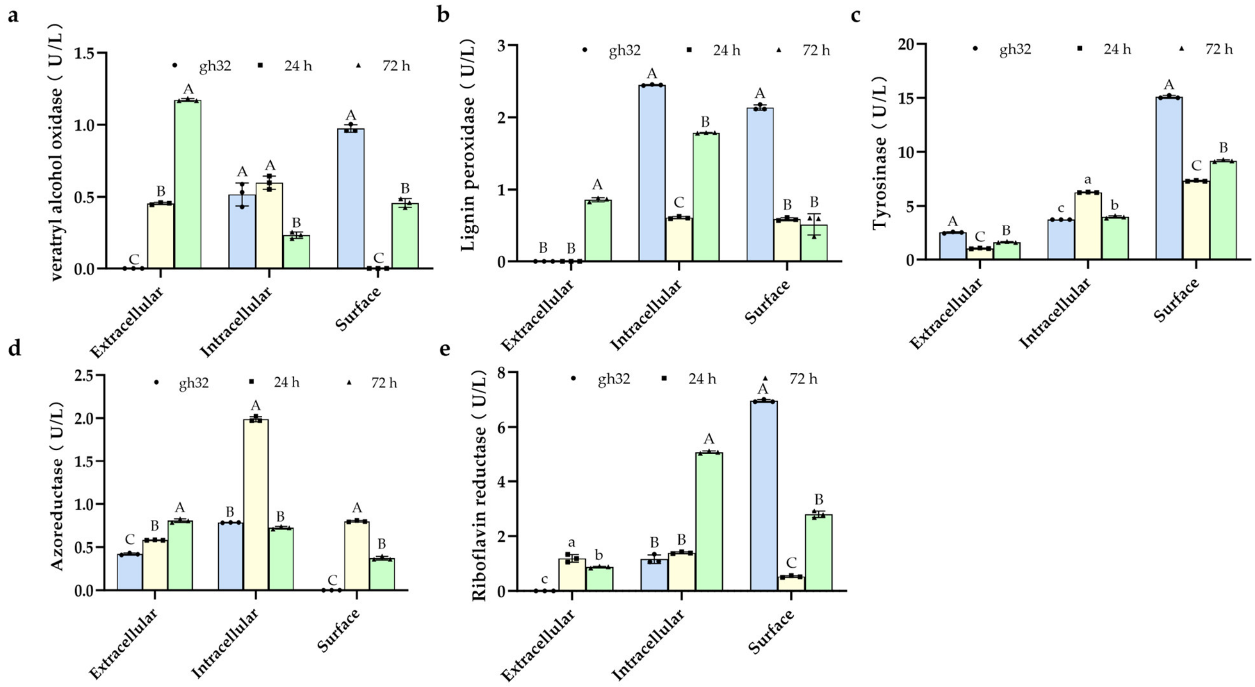

Figure S6. Changes in enzyme activities of strain gh32 for RB5 decolorization. (a) veratryl alcohol oxidase; (b) lignin peroxidase; (c) tyrosinase; (d) azoreductase; (e) riboflavin reductase. The data are analyzed by one way ANOVA with a Tukey multiple comparisons test using means of three experiments. Different lowercase letters indicate significant difference at $p < 0.05$. Different uppercase letters indicate significant difference at $p < 0.01$.

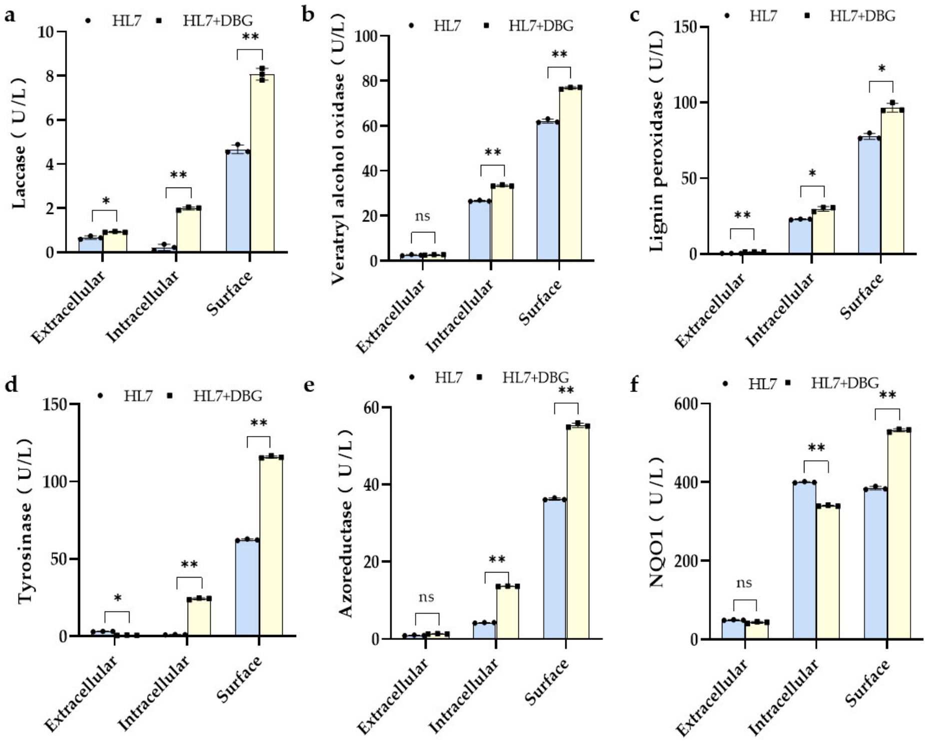

Figure S7. Changes in enzyme activity during DBG decolorization by strain HL7. (a) laccase; (b) veratryl alcohol oxidase; (c) lignin peroxidase; (d) tyrosinase; (e) azoreductase; (f) NADH:quinone oxidoreductase 1 (NQO1). The data are analyzed by a paired-sample t-test using means of three experiments. * indicates significant difference at $p < 0.05$. ** indicates significant difference at $p < 0.01$.ns indicates no significant difference.

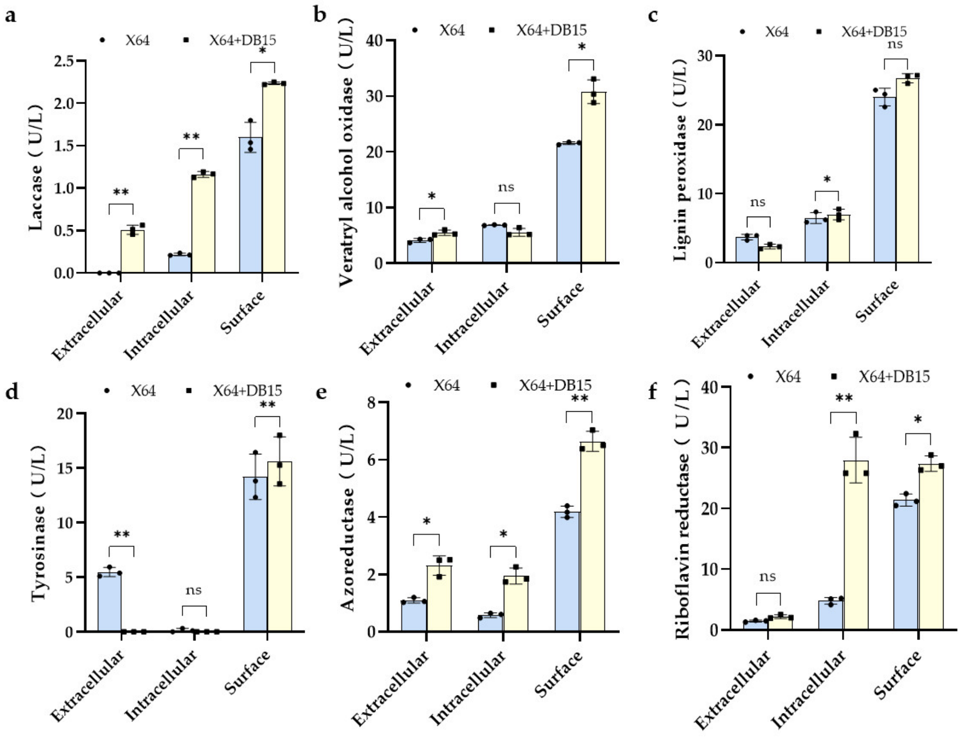

Figure S8. Enzyme activity profiling of bacterial strain X64 during Direct Blue 15 (DB15) degradation. (a) laccase; (b) veratryl alcohol oxidase; (c) lignin peroxidase; (d) tyrosinase; (e) aAzoreductase; (f) riboflavin reductase.The data are analyzed by a paired-sample t-test using means of three experiments. * indicates significant difference at $p < 0.05$. ** indicates significant difference at $p < 0.01$.ns indicates no significant difference.

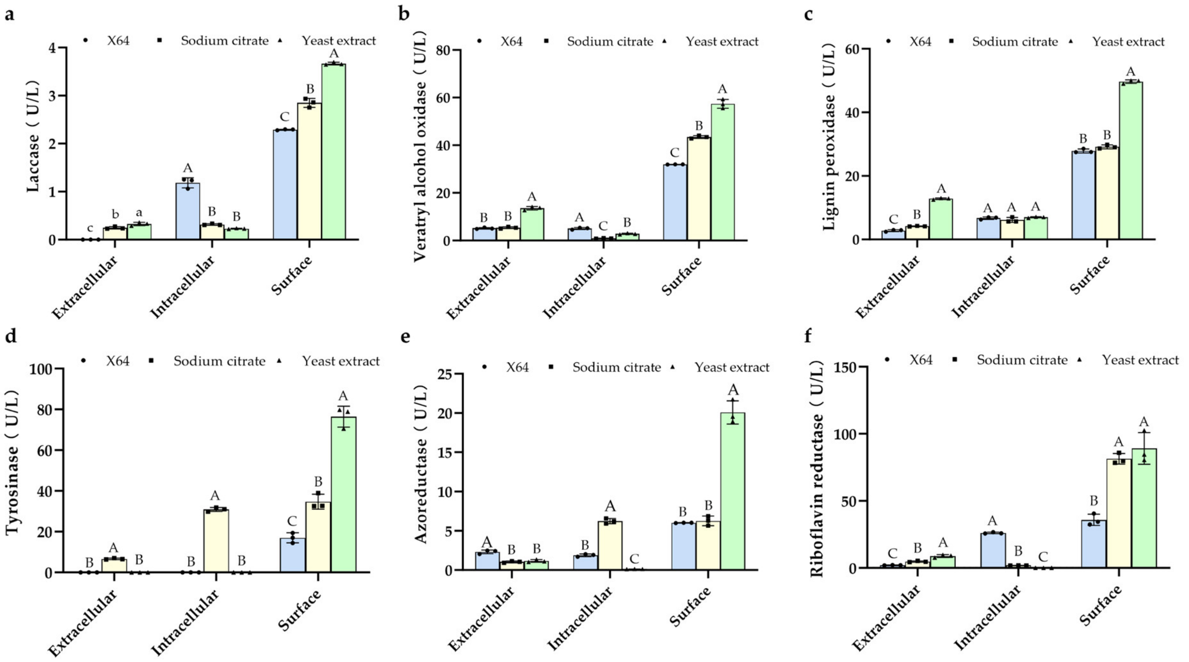

Figure S9. Enzyme activity profiling added substrates of bacterial strain X64 during direct blue 15 (DB15) degradation (a) laccase; (b) veratryl alcohol oxidase; (c) lignin peroxidase;(d) tyrosinase; (e) azoreductase; (f) riboflavin reductase. The data are analyzed by one way ANOVA with a Tukey multiple comparisons test using means of three experiments. Different lowercase letters indicate significant difference at $p < 0.05$. Different uppercase letters indicate significant difference at $p < 0.01$.

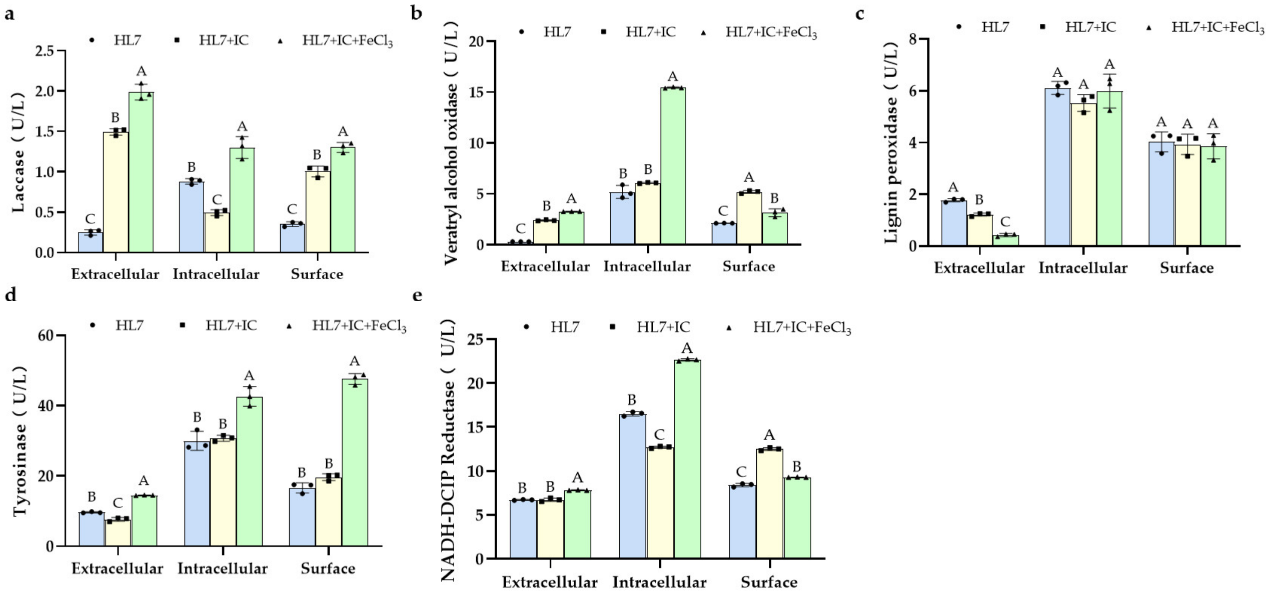

Figure S10. The effect of $FeCl_3$ on laccase activity during indigo carmine decolorization by strain HL7. (a) laccase; (b) veratryl alcohol oxidase; (c) lignin peroxidase; (d) tyrosinase; (e) NADH-DCIP reductase (NDR). The data are analyzed by one way ANOVA with a Tukey multiple comparisons test using means of three experiments. Different uppercase letters indicate significant difference at $p < 0.01$.
